# Tool-tissue force segmentation and pattern recognition for evaluating neurosurgical performance

**DOI:** 10.1038/s41598-023-36702-3

**Published:** 2023-06-13

**Authors:** Amir Baghdadi, Sanju Lama, Rahul Singh, Garnette R. Sutherland

**Affiliations:** Project neuroArm, Department of Clinical Neurosciences, Hotchkiss Brain Institute University of Calgary, Calgary, AB Canada

**Keywords:** Health care, Health care economics, Surgical oncology, Scientific data, Software, Statistics

## Abstract

Surgical data quantification and comprehension expose subtle patterns in tasks and performance. Enabling surgical devices with artificial intelligence provides surgeons with personalized and objective performance evaluation: a virtual surgical assist. Here we present machine learning models developed for analyzing surgical finesse using tool-tissue interaction force data in surgical dissection obtained from a sensorized bipolar forceps. Data modeling was performed using 50 neurosurgery procedures that involved elective surgical treatment for various intracranial pathologies. The data collection was conducted by 13 surgeons of varying experience levels using sensorized bipolar forceps, SmartForceps System. The machine learning algorithm constituted design and implementation for three primary purposes, i.e., force profile segmentation for obtaining active periods of tool utilization using T-U-Net, surgical skill classification into *Expert* and *Novice*, and surgical task recognition into two primary categories of *Coagulation* versus *non-Coagulation* using FTFIT deep learning architectures. The final report to surgeon was a dashboard containing recognized segments of force application categorized into skill and task classes along with performance metrics charts compared to expert level surgeons. Operating room data recording of > 161 h containing approximately 3.6 K periods of tool operation was utilized. The modeling resulted in Weighted F1-score = 0.95 and AUC = 0.99 for force profile segmentation using T-U-Net, Weighted F1-score = 0.71 and AUC = 0.81 for surgical skill classification, and Weighted F1-score = 0.82 and AUC = 0.89 for surgical task recognition using a subset of hand-crafted features augmented to FTFIT neural network. This study delivers a novel machine learning module in a cloud, enabling an end-to-end platform for intraoperative surgical performance monitoring and evaluation. Accessed through a secure application for professional connectivity, a paradigm for data-driven learning is established.

## Introduction

Incorporating artificial intelligence (AI) powered by cloud connectivity to aggregate data in and across operating rooms (OR) offers an objective tool for systematic feedback on the optimal use of medical devices and systems. This is important for improving the safety of surgery and utilizing digital innovation towards standardization of patient care. Implementing AI through sensor-enabled and data-driven surgical devices can transform traditional and subjective training based on apprenticeship into an objective and non-intimidating paradigm^1^. Context-aware assistance by surgical phase recognition can further facilitate and improve the training process through particularized analytic feedback on the performance of surgery^2^. As a new frontier in surgical coaching, surgical data science can be defined through novel frameworks involving collection, structuring, analysis, and modeling of such data^3,4^.

Machine learning algorithms in surgery, while early, may enhance care in various pathologies, including epilepsy, brain tumors, spine lesions, and cerebrovascular disorders^5^. Sensor-driven data can be used to accurately capture surgeon dexterity and technical skill, using meaningful features extracted from surgical maneuvers and workflow. This, in turn, would help provide a quantitative feedback metric during a graduated surgical training period. The movement of instruments has been used in the past as a kinematic measure of performance and skill discrimination in a laboratory environment^6–8^. For skill evaluation, a deep learning-based instrument tracking system based on surgical videos has been implemented, which is compliant with Objective Structured Assessment of Technical Skill (OSATS) and Global Evaluative Assessment of Robotic Skill (GEARS) manual metrics^9^. Surgical skill assessment and navigation in colorectal surgery can be facilitated through forceps type and object recognition on video data^10^. Additionally, the use of motion features extracted from video temporal pattern analysis led to the categorization and analysis of surgical actions^11,12^. A comprehensive review of surgical skill analysis literature has also been published^13^. The manuscripts included in this review used kinematic (61%) and video (29%) data, with limited attention to tool-tissue forces^14,15^. The ML models used herein were Artificial Neural Networks (ANN), Hidden Markov Models (HMM), and Support Vector Machines (SVM), all with higher accuracies than 80%. Their findings, however, were limited in data from real-life surgery (12%), as well as the lack of a framework application for providing surgeons with interpretable and clinically relevant feedback.

Among the sensory data, kinesthetic force feedback, i.e., concerning the reconstruction of the human sense of touch by activating muscular mechanoreceptors, is eminent. This type of force can have implications for surgical outcomes, e.g., non-optimal force application leading to tissue damage or prolonged surgical times^1,16,17^. In various studies, grip force was used as a metric for assessing surgical skill^6,18^. Instrument force analysis showed a lower force level in experienced surgeons than novices when performing dry laboratory exercise^6^. Furthermore, a regression analysis for automated skill evaluation based on contact force with task materials, robotic instrument accelerations, and task completion time was also performed^18^. The findings were in agreement with the manual GEARS metric. In addition, the combination of visual signals with force feedback has been shown to enhance tissue characterization^19^ with lower force peak magnitudes leading to significantly lower tissue trauma and surgical error rates^20^. Previous studies while successful in their respective goals, never focused on performance evaluation based on surgical tasks, e.g., coagulation as a paramount aspect of vascular surgery, using a single modality data from tool-tissue interaction, i.e., forces^16^.

Here we present an original machine learning framework, i.e., from data ingestion, analytics, and machine learning, to insights, for information extraction using a data-rich environment enabled by a sensorized bipolar forceps coupled to an intelligent software platform, the SmartForceps System^1,16,21–24^. The medical grade SmartForceps are sterilized between each procedure following the standards approved by regulatory bodies and the Central Sterilization and Reprocessing Department. For regulatory approval, we have demonstrated that each SmartForceps withstands multiple cycles of sterilization without impacting the instrument’s sensors, i.e., altering the calibration^1,25^. This novel framework builds upon our recent work on a data-enabled surgical performance dashboard, now creating an automated analytical platform. The work leverages sensor-based technology whereby evolving AI systems complement the way surgery is performed and taught. The modeling efforts encompass deep learning architectures and data analytics for surgical skill classification between *Expert* and *Novice*, and the recognition of a critical neurosurgical task, i.e., *Coagulation*, to improve granularity in performance feedback. Such analytics on surgical performance and comparison to the gold standard can be reviewed in an interactive environment, i.e., the *Expert Room*. This study offers new opportunities within an objective and sensor-driven surgical performance tracking and analytics model, towards improved learning and safety of surgery.

## Materials and methods

### Data recording

The SmartForceps System (developed at Project neuroArm, University of Calgary, Calgary AB, Canada) allows real-time display and recording of tool-tissue force data during surgery. Surgical tasks were categorized into: (1) *Coagulation* (cessation of blood loss from a damaged vessel), (2) *non-Coagulation* with sub-categories of (a) *Dissection* (cutting or separation of tissues), (b) *Pulling* (moving and retaining tissues in one direction), (c) *Retracting* (grasping and retaining tissue for surgical exposure), and (d) *Manipulating* (moving cotton or other non-tissue objects), which were identified following expert approval of cumulative data reviews. The audiotaped voice of each surgeon accompanied force recordings, which indicated the periods of force application and specific task names. This information facilitated the labeling process for each force segment, creating a supervised dataset for the machine learning models. The study was approved by the Conjoint Health Research and Ethics Board of the University of Calgary, Calgary, AB, Canada (REB19-0114), with the technology approved by Health Canada (ITA 329,641 Class II, 2021). Details on technology development, pre-clinical and clinical use have been previously published^16,21–23,26,27^. Informed electronic and verbal consent was obtained from participating surgeons per the REB, which included a waiver of informed patient consent by the Institutional Review Board at the University of Calgary. The surgical team adopted the SmartForceps system in place of the conventional bipolar forceps with the added advantage of real-time tool-tissue force measurement, display, and recording. Adult patients undergoing elective surgical treatment for various intracranial pathology were included in this prospective study (under the supervision of the senior author as the staff surgeon). Emergency neurosurgical procedures and the pediatric population were excluded. No identifiable patient information is included in the manuscript and methods were performed in accordance with the relevant guidelines and regulations for human experimental studies and Declaration of Helsinki.

The data framework included a HIPAA and PIPEDA Compliant Cloud architecture for retaining and processing the intraoperative de-identified data through a Cloud platform (Microsoft Azure, Microsoft USA) with secure authentication through organizational credentials. In addition, an installable web/mobile application was developed to monitor the force-related data/features, which is available at smartforceps-app.azurewebsites.net.

### Workflow architecture

**Figure 1:**
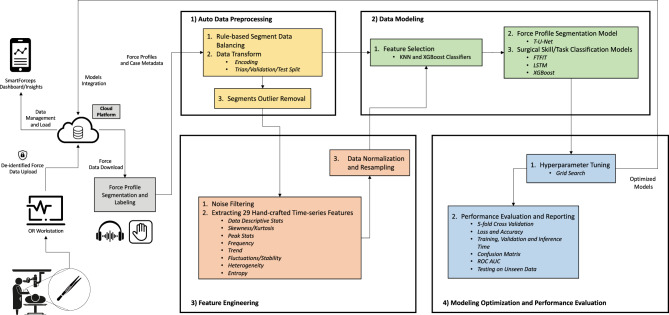
Workflow architecture of SmartForceps platform from data recording to modeling and visualization. Forces of tool-tissue interaction along with de-identified case information were uploaded to a HIPAA-compliant data storage and analytics platform. Force data were manually segmented and labeled by listening to the surgeon’s voice recordings, where surgeon names, surgical tasks, and important incidents were narrated. The AI modeling architecture included Auto Data Preprocessing (e.g., Data Balancing, Outlier Removal, Data Transformation, etc.), Feature Engineering, Data Modeling (T-U-Net for force profile segmentation (T-U-Net: Time-series-U-Net); XGBoost, LSTM and FTFIT (Force Time-series Feature-based InceptionTime) for pattern recognition), and Modeling Optimization and Performance Evaluation, which were integrated into the cloud platform to generate performance evaluation reports to the surgical team. A detailed description of selected processes in the figure has been described in the Supplementary Materials. Visualization was created in Microsoft PowerPoint version 16.49 with the icons obtained from a Google search: e.g., https://www.iconfinder.com.

To quantify the behavior of force profiles for pattern recognition and performance analysis, we developed machine learning models for segmenting and recognizing the patterns of intra-operative force profiles. The models make no assumption about the underlying pattern in force data and hence are robust to noise*.* The framework enables modeling a complex structure in our non-stationary time-series data, where data characteristics including mean, variance, and frequency change over time. Figure 1 shows the workflow architecture from data recording to modeling and visualization.

### Force profile segmentation

The force data points labeled as ON or OFF were included for analysis after applying rule-based data balancing (Figure S1, detailed in Supplementary Materials). To transform data to a normal distribution, i.e., Gaussian with zero mean and unit variance, and to eliminate the dominant effect of larger variance in a specific signal, feature normalization of the left and right prong force was performed by removing the mean and scaling to unit variance. This allowed standardization of corresponding values. Data preparation comprised of normalization and reshaping into windows of 224 data points which ended up with approximately 5.9 K resampled force windows. Following this, the segment labels were encoded, and a one-hot encoding schema was implemented. Finally, a 80% (20% validation)—20% split with a random seed was performed to split the data into training-validation and testing samples. A custom-designed U-Net (T-U-Net: Time-series-U-Net; U-Net is a dominant model for image segmentation^28^) model was trained and implemented that consisted of a convolutional encoder and decoder structure to capture the properties and reconstruct the force profile. Grid search was performed for hyperparameter tuning (Figures S2, S3, detailed in Supplementary Materials).

### Surgical force pattern recognition

#### Data pre-processing for surgeon skill classification

Segmented training data with binary *Expert* and *Novice* labels were included in this phase. To fortify prediction power in force data, a total of 29 hand-crafted features (Table S1) that can capture the behavior of surgical force time-series data and were analyzed in our previous study^1^ were calculated for each window of 200 data points and were added as the third signal to a deep learning model after a feature selection process (Supplementary Materials Tables S2, S3). Our data curation pipeline performed time-series based feature extraction on the segmented data after noise reduction^1^. The normalization process transformed the feature data into a Gaussian distribution with zero mean and unit variance and resampling to match the force data window size of 200 points with *ratio to maximum* as the order of spline interpolation and *edge* mode for the boundary data imputation. The normalized and reshaped data created 3.6 K resampled force segment windows (1766 *Novice* and 1859 *Expert* segments), which were encoded using a one-hot vector and a random split into training-validation, i.e., 80% (20% validation), and testing, i.e., 20%, samples.

#### Data pre-processing for surgical task recognition

In this phase, the main surgical task of *Coagulation* was considered as a data label to be distinguished from other tasks. Similar to the skill classification model, the 29 hand-crafted features (Table S1) were fed into the neural network after being calculated over 200 data point window, proper noise reduction, outlier removal, normalization, and resampling, and feature selection (Supplementary Materials Tables S4, S5). The processed force segments comprised of 2 K samples (1170 force segments of Coagulation and 915 segments of *non-Coagulation* (Manipulation = 323, Pulling = 316, Retracting = 149, and Dissecting = 127)) with one-hot encoding format having 64% training, 16% validation, and 20% testing samples.

#### Model implementations

Two deep learning and a baseline model were created to classify surgeon experience levels (i.e., *Novice* and *Expert*) and activity recognition while performing a specific task (i.e., *Coagulation* and *non-Coagulation* (e.g., *Pulling*, *Manipulation*, *Dissecting*, and *Retracting*)). A deep neural network model for time series classification based on InceptionTime^29^, i.e., FTFIT (Force Time-series Feature-based InceptionTime), was developed to obtain the learned features. This, together with engineered features described above, was used in a logistic regression-based surgeon experience classification. A second deep learning model based on an LSTM neural network for time-series-based surgeon activity and experience recognition was used. These models followed a baseline XGBoost classifier that used the hand-crafted features (details of the modeling and results are in the Supplementary Materials Figures S11, S12 and S18, S19). Further details on model characteristics and hyperparameter tuning are available in Supplementary Materials (Figures S4, S5, S6).

### Modeling evaluations

For all models, summary including the type, shape, and parameter counts for each layer; loss and accuracy values for both training and validation data in each epoch; classification report including fivefold cross-validation accuracy, selected model (through grid search on validation loss) testing accuracy (sensitivity and specificity), average precision, recall, weighted F1-score, and area under the curve (AUC) for receiver operating characteristic (ROC), and precision-recall curves during validation and testing with the corresponding charts and graphs were generated. Model training was performed using a workstation with Intel Core i9-9820X (10 cores, 4.20 GHz turbo) CPU, 2 × Titan RTX with NVLink GPU, and 64 GB memory taking approximately 0.7 h for the training and validation of data segmentation and 0.4 h for skill classification and task recognition models.

## Results

Tool-tissue interaction force data from 50 neurosurgery procedures of adult tumor resection (30 males/20 females, mean (SD) age: 54.7 (14.1)) between November 2019 and October 2020, including meningioma (n = 10), glioma (n = 10), schwannoma (n = 15), and hemangioblastoma (n = 3) (+ 12 other cases, e.g., trigeminal neuralgia/hemifacial spasm, cavernous angioma, etc.) was employed. The cases were performed by 13 surgeons, i.e., one *Expert* with 30 + years of experience and twelve *Novice* surgeons, including residents with post-graduate years (PGY) ranging across three levels of 1–2 (n = 4), 3–4 (n = 3), and > 4 years (n = 4), and one fellow.

### Force profile segmentation

Point-wise data classification as *ON* and *OFF* regarded as segments of force data through T-U-Net model showed the best results for 0.001 learning rate, 16 as filter size, moving window size of 224, and batch size of 128. The mean inference time was 0.24 s, and the minimum validation loss value occurred at epoch 27 (Figure S7a) was 0.1046 (training loss = 0.0853). fivefold cross-validation results showed a mean (SD) accuracy of 0.95 (0.01). Macro-AUC of ROC was 0.99 and when testing the model, accuracy was 0.95 (F1-score: 0.96 for class *ON*, and 0.95 for class *OFF*, weighted value = 0.95) (Table 1). Detailed results are illustrated in Figure S7, S8, S9, S10 (Supplementary Materials).

**Table 1:** Combined best modeling performances for SmartForceps Machine Learning pipeline.

	SmartForceps machine learning pipeline step		
	Force profile segmentation	Surgical skill classification	Surgical task recognition
Model name	T-U-Net	FTFIT	FTFIT
Best performing hyperparameters	learning rate = 0.001 filter size = 16 window size = 224 Batch size = 128	learning rate = 0.001 depth size = 6 window size = 200 Batch size = 128	learning rate = 0.01 depth size = 12 window size = 200 Batch size = 128
Mean inference time (s)	0.24	0.24	0.20
Mean (SD) 5-Fold cross-validated accuracy	0.95 (0.01)	0.73 (0.03)	0.79 (0.07)
Testing accuracy	0.95	0.71	0.82
Testing sensitivity/recall	0.97	0.78	0.90
Testing specificity	0.94	0.66	0.78
Average precision score	0.98	0.81	0.89
Testing weighted F1-score	0.95	0.71	0.82
Respective AUC	0.99	0.81	0.89

### Surgical skill classification

**Figure 2:**
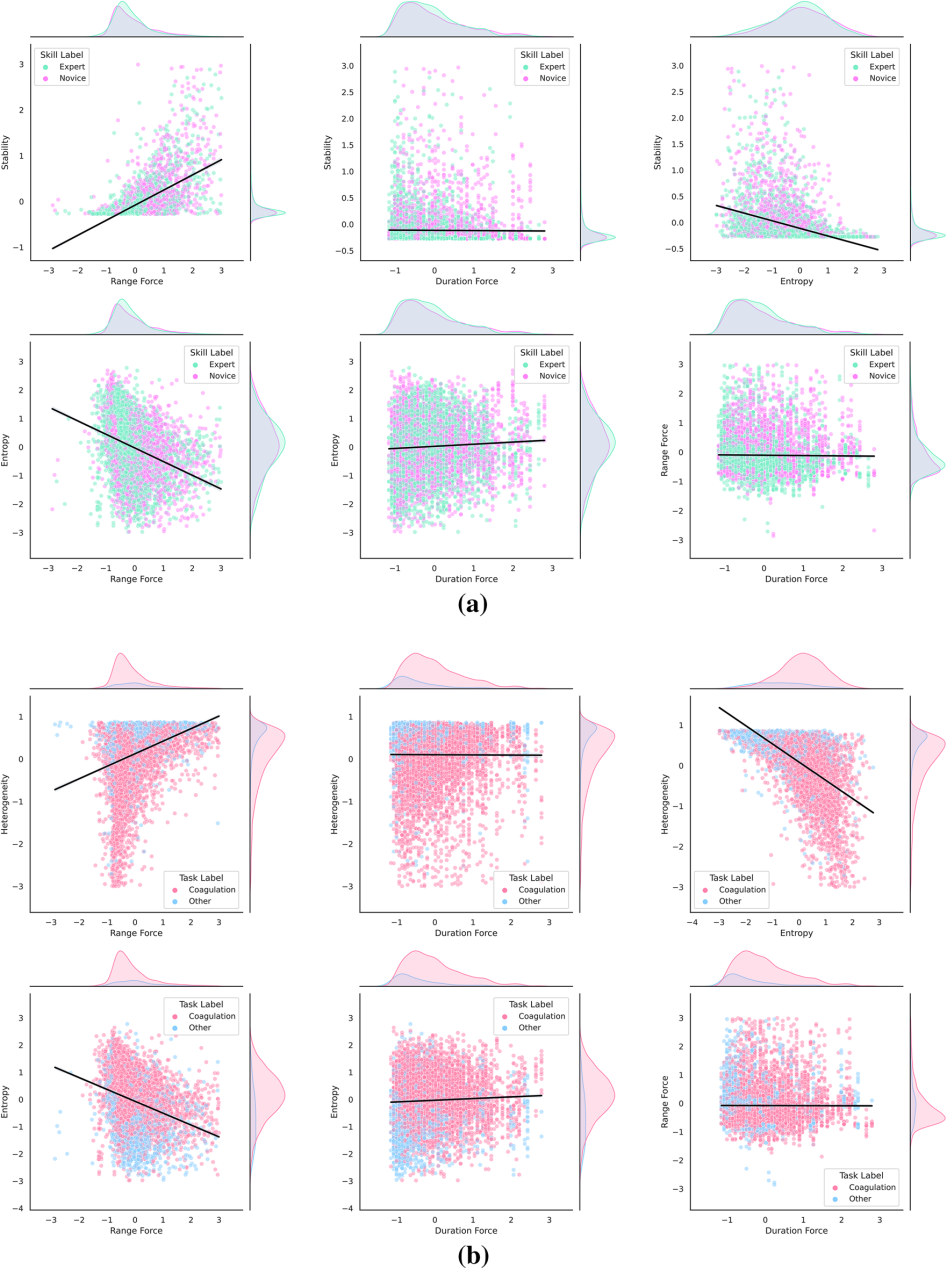
Correlation and distribution plots for standardized *subset 1* hand-crafted features with their respective class labels used in the Skill Classification and Task Recognition models.

**Figure 3:**
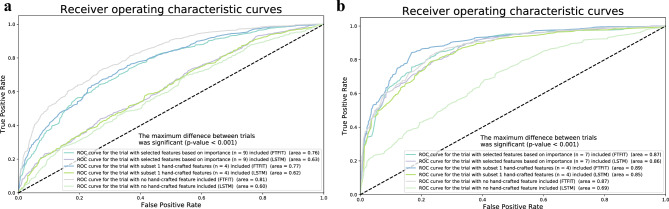
Performance comparison between LSTM and FTFIT models with hand-crafted feature combinations in surgical skill prediction and task recognition. Different combinations of hand-crafted features, i.e., no feature, selected set of features identified through KNN and XGBoost feature importance ranking, and a subset of features (Duration Force, Range Force, Entropy, and Heterogeneity; which were consistent with the features presented in SmartForceps performance dashboard^1^) have been compared.

The overlapping distribution of features in *Expert* and *Novice* classes is an early sign for a sub-optimal performance of feature augmentation to the network (Fig. 2a). Time-series classification performed best in FTFIT with no hand-crafted features added to the network (AUC = 0.81; *p* value < 0.001) (Fig. 3a). The model was characterized by a learning rate of 0.001 and a network depth size of 6, moving window size of 200, and batch size of 128. Testing time for each sample occurred in an average of 0.24 s, and the model reached minimum validation loss at epoch 66 (out of 100 epochs) (validation loss = 0.5285 and training loss = 0.4841 (Figure S14a)). Macro-AUC of ROC was 0.81 and while testing the model for unseen instances of force data, the accuracy was 0.71 (mean (SD) value of fivefold cross-validated accuracy was 0.73 (0.03)) with F1-score of 0.71 in both *Expert*, and *Novice* classes (weighted value = 0.71) (Table 1). Detailed results are available in Figures S14, S15, S16, S17 (Supplementary Materials).

- **Skill classification model** The figure shows the shape of standardized (Gaussian with zero mean and unit variance) data distribution for each skill, i.e., normal with a low tendency of negative skewness in Entropy, normal with a low tendency of positive skewness in Range Force, negative skewed in Heterogeneity, and positively skewed in Duration Force. In addition, a positive correlation in Stability vs. Range Force and a negative correlation in Entropy vs. Range Force and Stability vs. Entropy was observed. Note: data visualization was created after outlier removals of Z-score < 3 across the samples.
- **Task recognition model** The shape of standardized data distribution for coagulation vs. other tasks, i.e., normal in Entropy, negatively skewed in Heterogeneity, and positively skewed in Duration Force and Range Force has been shown. In addition, a positive correlation was noted in Heterogeneity versus Range Force and a negative correlation in Entropy versus Range Force and Heterogeneity versus Entropy. Note: data visualization was created after outlier removals of Z-score < 3 across the samples.

**(a) Surgical skill prediction** The best performing model was FTFIT with no hand-crafted features added to the network showing an AUC = 0.81 (*p* value < 0.001).

**(b) Surgical task recognition** FTFIT with *subset 1* of the hand-crafted features (n = 4) added to the network was among the best performing models with an AUC = 0.89 (*p* value < 0.001).

### Surgical task recognition

Performance of task recognition for *Coagulation* and *non-Coagulation* (after a random selection of 0.5 *Coagulation* segments for data balancing) using FTFIT with *subset 1* of the hand-crafted features (n = 4) added (Fig. 2b) to the network was among the best results (AUC = 0.89; *p* value < 0.001) (Fig. 3-b). This model had a learning rate of 0.01, a network depth size of 12, a moving window size of 200, batch size of 128, and concluded with a mean inference time of 0.20 s. This model's minimum validation loss value occurred at epoch 46 (out of 150 epochs) with validation loss of 0.4002 and training loss of 0.3025 (Figure S21a). Macro-AUC of ROC was 0.89 and testing results showed 0.82 in accuracy with a mean (SD) fivefold cross-validated accuracy of 0.79 (0.07). (F1-score of the *Coagulation* class was 0.85 and for *non-Coagulation* it was 0.77; weighted average = 0.82) (Table 1). Detailed results are available in Figure S21, S22, S23, S24 (Supplementary Materials).

### End-to-end pipeline implementation

**Figure 4:**
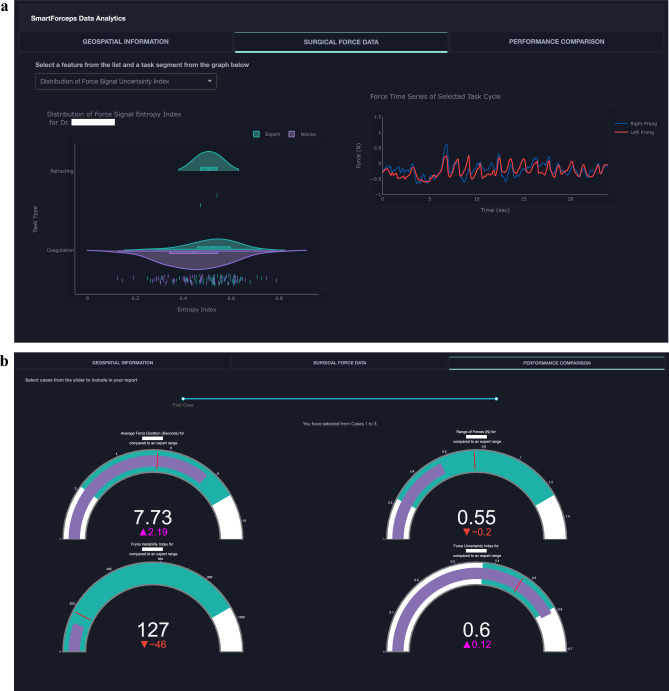
Overview of the processed data for three surgical procedures using SmartForceps machine learning modeling and recognition. This figure is a snapshot of the Surgical Force Data tab in the SmartForceps performance monitoring dashboard showing the final output of the data and analytics pipeline. The pipeline started from operating room data collection using dedicated software, continued with Microsoft Data Factory running analytics engine for preprocessing data and pattern recognition, and ended with the mobile/desktop performance monitoring dashboard.

Machine learning models were translated to resources and pipelines embedded in the cloud platform for *on-the-fly* analytics and feedback to surgeons. Final output for segmentation and skill/task recognition was visualized through comparative distribution plots and individual force profile segments as previously described^1^. Figure 4 shows the force profiles and performance report of a surgeon across 3 cases of brain tumor resection.

**(a) Surgical force data** In this representation, aggregative data distribution of both Expert-level (green violin plots) and Novice-level (purple violin plots) performance of the surgeon across the surgical tasks based on force Uncertainty Index (i.e., Entropy as a feature) selected from the dropdown menu (left column chart) is reported. The right column chart shows actual force profiles for the left (red time-series plot) and right (blue time-series plot) prong of SmartForceps.

**(b) Performance comparison** This figure shows performance comparisons (purple bar) of the surgeon compared to the *Expert* level surgeons (mean and standard deviation indicated as red mark and green area, respectively) after surgical-force-related feature extraction on segmented force profiles by T-U-Net. The gauge charts show the respective values for Average Force Duration, Range of Forces, Force Variability, and Force Uncertainty Indexes across 3 surgical procedures performed by the surgeon. In this graph, the representative surgeon gauge starts from zero as the baseline with the goal of reaching to the expert level values denoted by a red bar and green area. This surgeon had a higher average force duration (2.19 s more), lower range of forces (0.2 N less), lower force variability (46 points less), and higher force uncertainty (0.12 points more) compared to the average of expert data.

## Discussion

This work presents an original algorithm running in perpetuity *behind-the-scenes* in the OR as a data-enabled virtual surgical assistant in real-world settings^30,31^. Built upon the time-series data obtained using SmartForceps, a step-by-step process was developed to establish unique machine-learning models custom-tailored for real-time credible performance feedback and interconnectivity in the OR. Indeed, such automated analytics based on tool-tissue interaction force provided a holistic view of combinatorial elements shaping surgical skill, e.g., tool-tissue forces, force profile, motion, hand–eye coordination, etc., all of which may contribute to surgical finesse^1,32^.

In training a machine learning framework, any data imbalance can pose a challenge in the predictive models through bias toward the majority class. This problem was mitigated through *data elimination*, i.e., force block removal during device-idle periods in force profile segmentation, and random 50% sampling of force segments in the high frequent task of *Coagulation*. Since the time-series segmentation model provides a point-wise classification of *ON*/*OFF* instances, post-processing analytics are necessary for the production phase, including extracting continuous *force*-*ON* blocks meeting data length requirements and reconciling the noise-driven discontinuity in the identified segments. Production pipeline incorporated data factories, functions, and REST APIs that, following the upload of OR data into the cloud, served as personalized performance monitoring dashboard application^1,27^.

While annexing hand-crafted features to neural networks for skill recognition and task classification models was preferred, the output can imply dual inference. Following extensive trials, it was evident that a selected feature-set incorporation would reduce performance in skill classification (AUC reduces from 0.81 to 0.76 in FTFIT when using the window size of 200). On the other hand, the combination of replicated performance features in the SmartForceps monitoring dashboard^1^, e.g., Duration Force, Range Force, Entropy, and Heterogeneity, were among the best performing combinations in deep learning models (performance range from AUC = 0.85 to 0.89 in task recognition models using LSTM or FTFIT). This showed the importance of application-specific optimal modelling and its validation for usage in the real world. In machine learning models with limited data, overfitting occurs frequently, and the baseline models indicate such a phenomenon as shown in the Supplementary Materials with the differences seen between training and testing accuracies. To mitigate overfitting, grid search for model fine tuning and early stopping based on validation loss were implemented. A fivefold cross-validation was performed to assess the final performance of the model based on the best hyperparameters. The results showed good matching between testing and mean (SD) cross-validated accuracies for segmentation (0.95 vs. 0.95 (0.01)) and skill classification (0.71 vs. 0.73 (0.03)), but higher variabilities for task recognition (0.82 vs. 0.79 (0.07)). However, an accuracy of 0.7 is the minimum value that is covered across all the cross-validated models.

Although included feature sets went through a normalization process before circulating in the deep neural networks, some of these features inherent variability and noise-prone characteristics (e.g., Spikiness and Coefficient of Variance with very low and high variabilities, respectively) would negatively affect a time-series profile descriptor. The distribution of feature values in Fig. 2a, despite Fig. 2b for task categories, showed high similarity across skill levels. This is, to some extent, reflected in performance comparison between the two pattern recognition efforts, i.e., task recognition has a better performance than skill classification. This suboptimal performance of surgical skill classification can be explained through a statistical analysis of the factors underlying this model. Our analysis showed that the mean (SD: Standard Deviation) for *Force Duration* in *Coagulation* was 12.1 (7.2) seconds (i.e., around 58% higher than the average of completion time in other tasks: two-way ANOVA test *p* value < 0.001), however, this measure comparison across Expert and Novice groups was 12.2 (7.2) versus 12.1 (7.3) only 0.8% difference. Similar behavior was observed for Minimum Force (Task Classes *p* value = 0.1; Skill Classes *p* value < 0.001), Force Distribution Skewness (Task Classes *p* value = 0.8; Skill Classes *p* value < 0.001), and Force Profile First Autocorrelation Zero (Task Classes *p* value = 0.9; Skill Classes *p* value < 0.001)^13^*.* A lower performance for skill classification has also been reported in previous studies where investigators showed a mean precision of 91% in detecting surgical actions, however, 77% when predicting surgical skills using deep learning on surgical videos ^6^. This may in part relate to *real-world scenario* whereby trainee surgeons perform only those tasks delegated by the attending based on their level of competency and comfort. In addition, this similarity of pattern can perhaps be attributed to the trainees following the mentor’s lead in our single institutional data. Including multi-institutional data with more distinctive patterns across mentor-trainee populations and procedures would help equip and enrich the machine learning framework with more granularity and diversity of incoming data, i.e., rating of skill proficiency, into the skill level classification model.

Of interest, the input time-series window size had an impact on modeling performances, i.e., AUC = 0.78 to 0.81 for the skill classification model and AUC = 0.87 to 0.89 for the task recognition model using the FTFIT network. This primarily related to the average duration time of a force segment, which was close to 10 s (200 data points considering the sampling rate of 20 Hz). Internalization of the FTFIT network for SmartForceps data modeling showed significant improvement compared to a widely used deep learning model, i.e., LSTM, for both skill recognition (AUC improvements from 0.60 to 0.81) and task recognition (AUC improvements from 0.69 to 0.89). This comparison with the baseline XGBoost model also showed an improvement in testing accuracy, i.e., from 0.65 to 0.71 in skill classification and from 0.81 to 0.82 in task recognition. Convolutional operations in FTFIT further allowed the local structure of force profile, e.g., line and curves, to be captured in bottom-layer neurons of the network, while various shapes, e.g., valleys and hills, in the top layers. Additionally, the speed performance, and scalability establish FTFIT as a suitable candidate for widespread use of the SmartForceps machine learning platforms^29^.

Efforts in utilizing AI for surgical monitoring and performance assessment have been initiated, mainly in surgical robotics, linking haptic feedback, robot kinematics, and clinical information such as operating time, blood loss, etc. to predict surgical outcomes as a measure of performance^33,34^. Similarly, surgical video-based localizing of surgical instrument’s trajectory and motion characteristics, using the Fast R-CNN model have been employed for estimation of performance monitoring^35^. For surgical task recognition, investigators have studied continuous kinematic data represented as strings for discriminative gesture discovery via relative occurrence frequency measured by comparative numerical statistics^36^. Low-level spatiotemporal features from video data, combined with a high-level segmental classifier based on a convolutional neural network integrating visual objects with temporal components have also been used^37^. Data-driven approaches have been developed for clinical decisions support. Multi-source preoperative and intraoperative data from a large number of surgical cases were used to predict postoperative complications^38^. Additionally, deep learning was used to forecast surgical duration in real-time for informed preoperative decisions^39^. Although one-to-one comparison of performance between studies was deemed unsuitable due to differing goals, a single modality, force data, has never been used to evaluate performance based on surgical tasks, such as coagulation as a vital component of vascular surgery.

## Limitations

This study was limited by the inclusion of only one Expert surgeon, which may affect the surgical skill classification model. As the technology currently spreads to other centers, it allows a diversified data collection from a variety of surgical teams. Clinical trials and retraining of models with larger numbers of surgical teams will be included in future studies.

## Conclusions

Here, using the SmartForceps technology, we have developed a unique *end-to-end data-enabled pipeline* that consolidates the concepts of immortalizing surgical skills. Perhaps this could be considered as a virtual assist hosted in a cloud platform enabling access beyond geographical or generational limits. Facilitating contemporary transition to competency-based surgical education and practices, sensor-driven technology, which allows a digital, quantifiable output, is deemed timely and necessary.

### Additional information

Supplementary figures and table are available for this paper through this link: https://github.com/smartforceps/ai_models/tree/main/supplementary-files.

## Data and code availability

Sample de-identified data and modeling codes are available at a GitHub repository: https://github.com/smartforceps/ai_models.

## Supplementary Information

Supplementary Information.
