# Supplementary material for: Tool-tissue force segmentation and pattern recognition for evaluating neurosurgical performance
Source: Sci Rep. 2023 Jun 13;13:9591. doi: 10.1038/s41598-023-36702-3 (PMC10264433; doi:10.1038/s41598-023-36702-3)
Supplement: Supplementary file 1 — Supplementary Information. [file 41598_2023_36702_MOESM1_ESM.pdf]

List of Supplementary Figures and Tables

Fig. S1: Pseudo code for rule-based data point filtering to balance *ON* and *OFF* data samples.

Fig. S2: Compact visualization of T-U-Net model architecture used for segmentation of force signals.

Fig. S3: The T-U-Net model architecture used for segmentation of force signals.

Fig. S4: Compact visualization of FTFIT model architecture used for classification of surgical skills and tasks using time-series of segmented force profiles.

Fig. S5: The FTFIT model architecture used force profile pattern recognition on time-series of segmented force profiles.

Fig. S6: The LSTM model architecture used as an experimental model for force profile pattern recognition on time-series of segmented force profiles.

Fig. S7: Accuracy and loss function values during training and validation steps for force profile segmentation using U-Net model.

Fig. S8: The summary prediction results for the segmentation model as binary classification problem using U-Net model.

Fig. S9: The prediction performance for the segmentation model through receiver operating characteristic curves.

Fig. S10: Illustrating the trade-off between true positive rate and positive predictive value in different probability thresholds in segmentation.

Fig. S11: Importance ranking of the hand-crafted features in skill classification using XGBoost modeling.

Fig. S12: Experimental results of skill classification with various learning rate and minimum child weight using XGBoost model.

Fig. S13: Permutation importance ranking of the hand-crafted features in skill classification using KNN modeling.

Fig. S14: Accuracy and loss function values during training and validation steps for force profile surgical skill pattern recognition using FTFIT model.

Fig. S15: The summary prediction results for surgical skill prediction using FTFIT model.

Fig. S16: The prediction performance for the skill classification model through receiver operating characteristic curves.

Fig. S17: Illustrating the trade-off between true positive rate and positive predictive value in different probability thresholds in the skill classification.

Fig. S18: Importance ranking of the hand-crafted features in task recognition using XGBoost modeling.

Fig. S19: Experimental results of task recognition with various learning rate and minimum child weight using XGBoost model.

Fig. S20: Permutation importance ranking of the hand-crafted features in task recognition using KNN modeling.

Fig. S21: Accuracy and loss function values during training and validation steps for force profile surgical task pattern recognition using FTFIT model.

Fig. S22: The summary prediction results for surgical task recognition using FTFIT model.

Fig. S23: The prediction performance for the task recognition model through receiver operating characteristic curves.

Fig. S24: Illustrating the trade-off between true positive rate and positive predictive value in different probability thresholds in the task recognition.

Table S1: List and description of hand-crafted features for the surgical pattern recognition models.

Table S2: Relative importance scores of features for skill classification with a threshold of 0.05 using XGBoost model.

Table S3: Relative importance scores of features for skill classification with a threshold of 0.03 using KNN model.

Table S4: Relative importance scores of features for task recognition with a threshold of 0.05 using XGBoost model.

Table S5: Relative importance scores of features for task recognition with a threshold of 0.015 using KNN model.

Methods

Force Profile Segmentation

- ***Rule-based Segment Data Balancing (Fig. 1, Section 1.1)***

  A rule-based data point filtering was applied to mitigate the problem of imbalanced data in *ON* and *OFF* conditions for the recorded force data. In fact, 93.7% of the force data points were labeled as *OFF* (among a total of 11.6 million records), meaning that inactive status constructs most operating room times for SmartForceps. The algorithm performed inactive state removal by eliminating the excessive idle time points when the rolling average with a window of 5 for the left and right prong forces was less than or equal to 0.3 (N). The points with overlapping *OFF* labels in both rule-based and manually labeled data were removed from the analysis (data size reduced to approximately 398K records) (Fig. S1). This data regularization method resulted in 54.4% in *ON* labels and 45.6% in *OFF* labels, making the segmentation labels balanced across the two classes.

```
>  for each (F_{r, idx} & F_{l, idx}) in F_s:
       if (MA_idx (F_r, window=5) & MA_idx (F_l, window=5) <= 0.3) & (init_seg_id_idx = 0):
           remove F_{r, idx} & F_{l, idx}
```

$F_{r,\ idx}$: idx^{th} data point of the right prong force profile.
$F_{l,\ idx}$: idx^{th} data point of the left prong force profile.
F_s: SmartForceps force profile time-series.
MA_{idx} (X, window=w): Moving average of time-series X with a window size of w at idx^{th} data point.
$init\_seg\_id_{idx}$: Initial segmentation ID (i.e., 0: OFF, 1: ON) at idx^{th} data point.

Fig. S1 | Pseudo code for rule-based data point filtering to balance *ON* and *OFF* data samples. A rule-based algorithm was designed to remove the excessive inactive time points when the rolling average with a window of 5 for the left and right prong forces were less than or equal to 0.3 (N). Data points with overlapping 0 (*OFF*) labels in both rule-based and manually labelled indices were removed.

- ***Force Profile Segmentation Model (Fig. 1, Section 2.2)***

  a. *T-U-Net*

A custom-designed U-Net (T-U-Net: Time-series-U-Net) model was implemented that consisted of a convolutional encoder and decoder structure to capture the properties and reconstruct the force profile ($\boldsymbol{X}_{in} \in \mathbb{R}^{S_0 \times i \times C}$: S_0 fixed-length segment interval each containing i data points through $C = 2$ channels for left and right prong) through a deep stack of feature maps. A mean-pooling-based classifier follows this on point-wise confidence scores for interval-wise time-series segmentation ($\boldsymbol{X}_{seg.} \in \mathbb{R}^{S \times K}$: S final segment intervals containing $K = 2$ segment classes, i.e., device ON/OFF). For the training parameters, we considered *Adam* as the optimizer, *Categorical Cross-Entropy* as the loss function, and *accuracy* and *validation loss* as the evaluation metrics for a random 20% subset of training data as the validation data. Grid search was performed over the learning rate, i.e., within [0.0001-0.1], T-U-Net filter values, i.e., within [16-128], and batch size, i.e., within [32-128] for hyperparameter tuning. The compact visualization of the model architecture is provided in Fig. S2, and the expanded view of the model created by https://netron.app is present in Fig. S3.

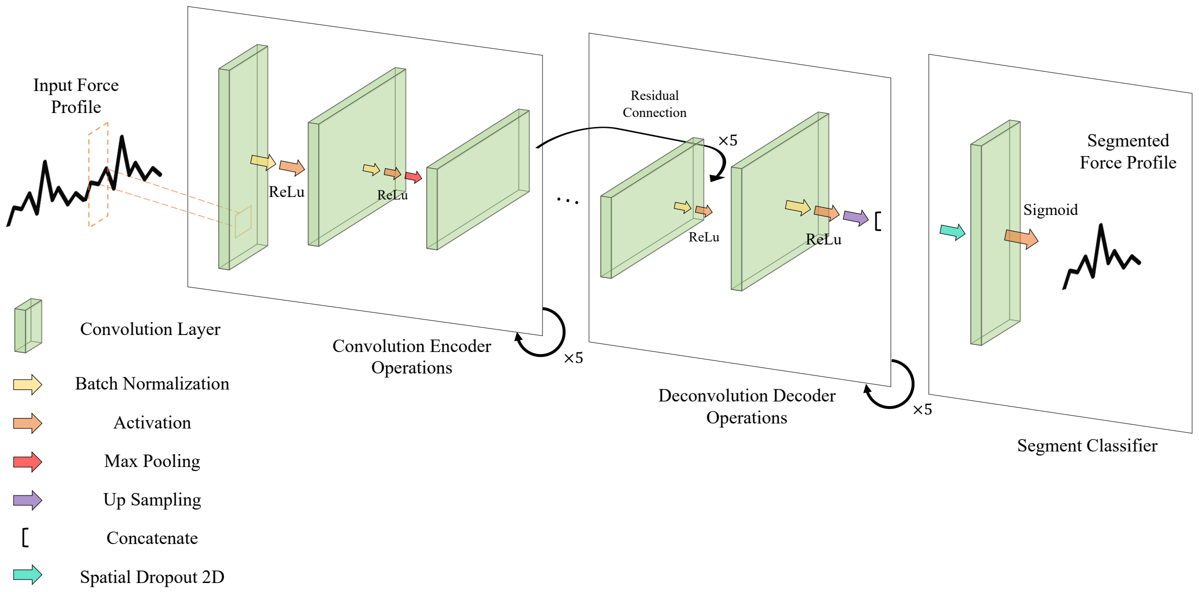

Fig. S2 | Compact visualization of T-U-Net model architecture used for segmentation of force signals. The network is comprised of different *convolutional encoder* and *decoder* operations with residual connection bypath and intermittent operations, e.g., *batch normalization*, *activation*, *max pooling*, *up sampling* and *concatenation layers*, and a final segment classifier with *spatial dropout* and *activation*. The visualization was created in Microsoft PowerPoint version 16.49 with the icons obtained from a Google search: e.g., https://www.iconfinder.com.

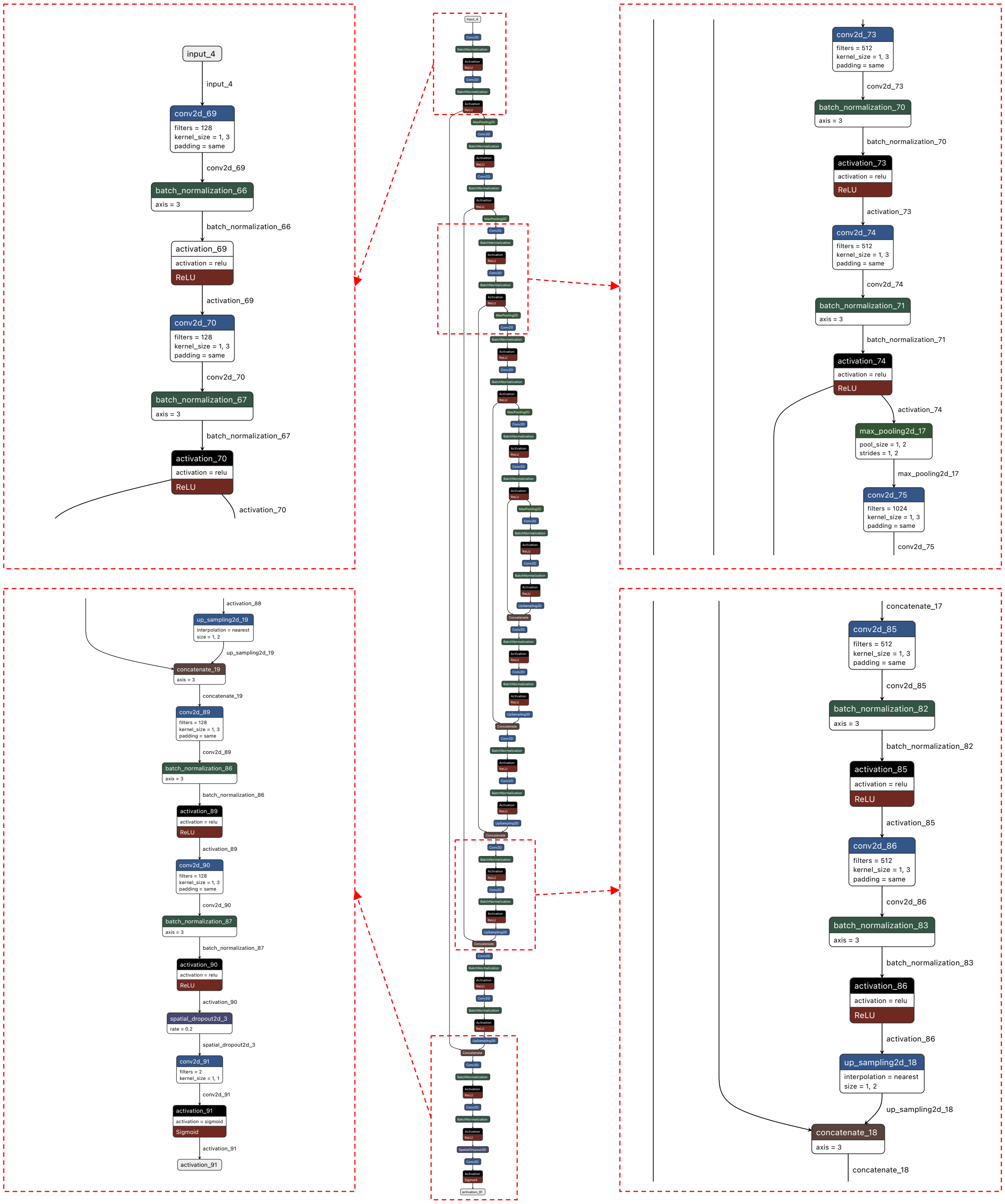

Fig. S3 | The T-U-Net model architecture used for segmentation of force signals. The graph shows detailed procedure names and attribute values for force profile segmentation model. The network comprised of different *convolutional encoder* and *decoder* operations with residual connection bypass and intermittent operations, e.g., *batch normalization*, *activation*, *max pooling*, and *concatenation layers*, and a final segment classifier with *activation*. The visualization was created in https://netron.app.

Surgical Force Pattern Recognition

- ***Surgical Skill Classification Model (Fig. 1, Section 2.3)***

    - ***FTFIT Model***

A model based on InceptionTime was developed, i.e., FTFIT (Force Time-series Feature-based InceptionTime), where force time-series related features were merged as new dimensions to the network and the architecture was optimized to fit the specifics of tool-tissue interaction forces in surgery. The input to the network was a segmented force time-series ($\boldsymbol{X}_{seg.} \in \mathbb{R}^{S \times C}$: S segment intervals over $C = 2$ channels of left and right prong data in SmartForceps). The network included multiple layers including, a *bottleneck layer* to reduce the dimensionality after a *max-pooling layer*, a stacked series of *convolutional layers* to learn the features, followed by a *concatenation layer*. Finally, the extracted features were fused into the network after *resampling* and *normalization* as a new dimension. The network's output was the probabilities of different classes, e.g., surgical proficiency scores or the task categories. As the evaluation metrics, we used *Adam* optimizer on *Categorical Cross-Entropy* along with a customized loss function (details in the supplementary codes), *accuracy* and *validation loss*. We applied grid search over the learning rate, i.e., within [0.001-0.1], and network depth, i.e., within [6-12] layers, input data window size, i.e., within [96-200], and batch size, i.e., within [32-128] for hyperparameter tuning. The compact visualization of the model architecture is provided in Fig. S4, and the expanded view of the model created by https://netron.app is present in Fig. S5.

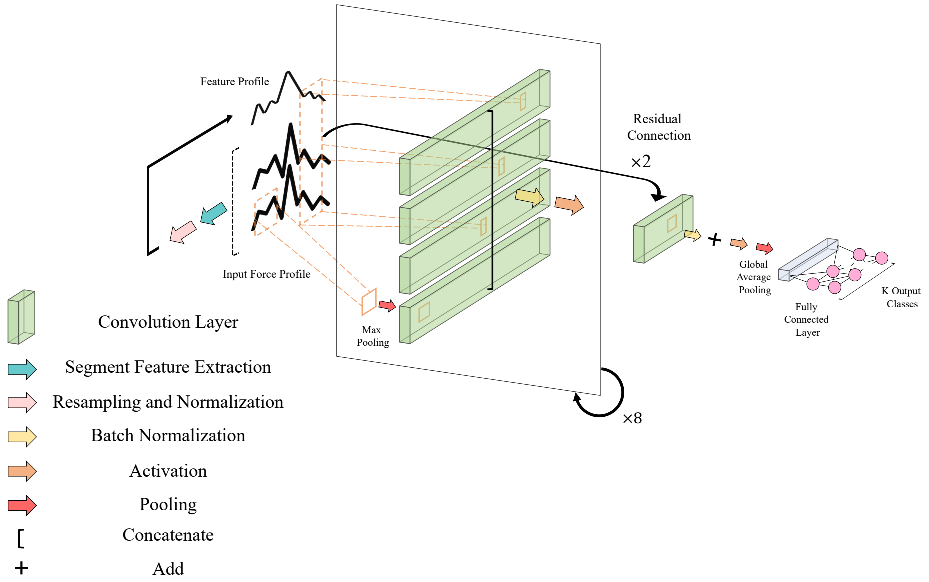

Fig. S4 | Compact visualization of FTFIT model architecture used for classification of surgical skills and tasks using time-series of segmented force profiles. The network included multiple layers, including a stacked series of *convolutional layers* to learn the features, followed by a concatenation *layer*, a *bottleneck layer* to reduce the dimensionality accompanied by a *max-*

pooling layer. As a new dimension, the extracted features were fused into the network after *resampling* and *normalization*. The last layer shaped the probabilities of different classes, e.g., surgical proficiency scores or the task categories. The visualization was created in Microsoft PowerPoint version 16.49 with the icons obtained from a Google search: e.g., https://www.iconfinder.com.

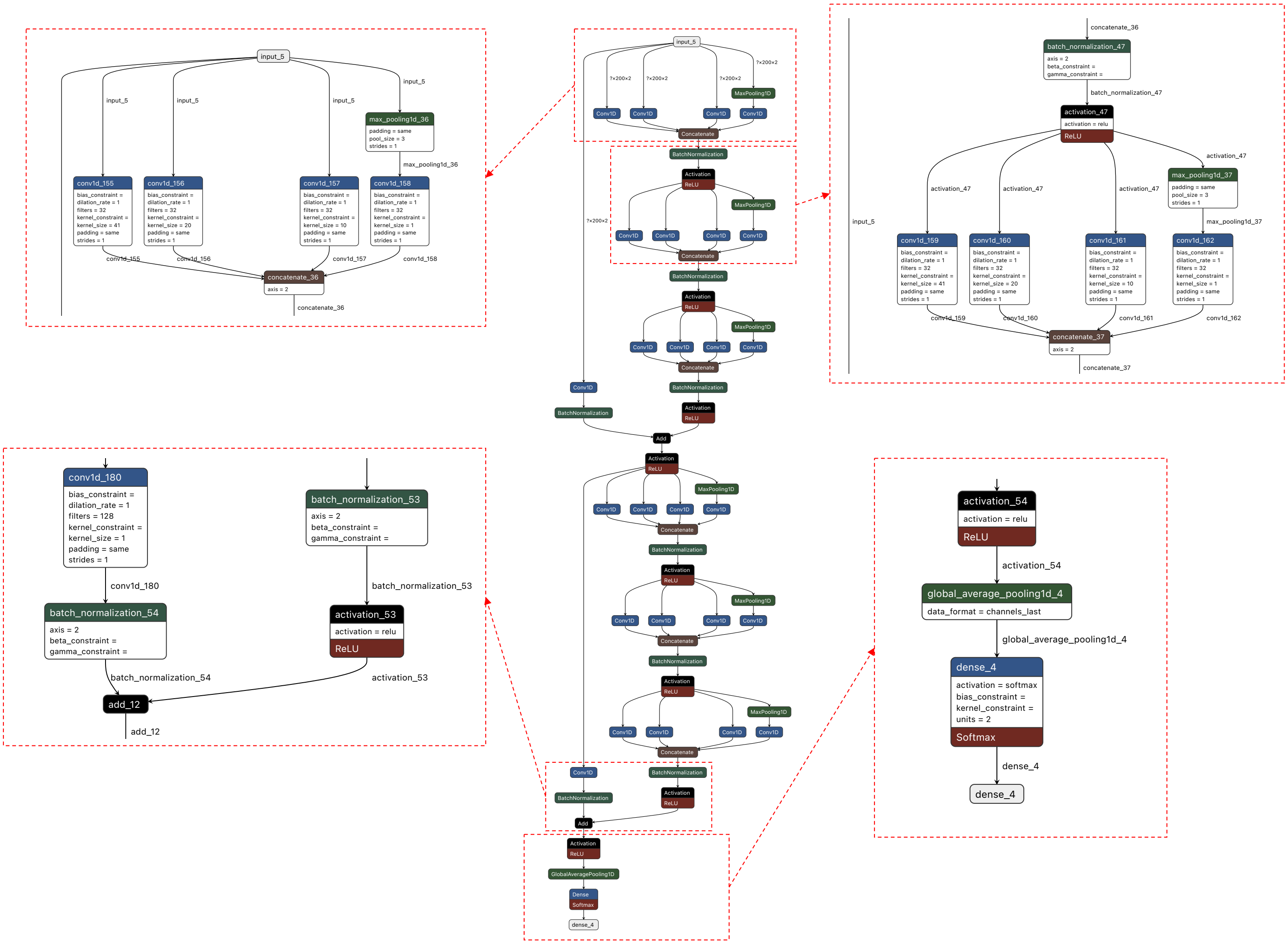

Fig. S5 | The FTFIT model architecture used force profile pattern recognition on time-series of segmented force profiles. The graph shows detailed procedure names and attribute values for skill classification model (depth size = 6). Note: the network for task recognition is not included in the report to avoid duplication. The network included multiple layers including a stacked series of *convolutional layers* to learn the features followed by a concatenation *layer*, a *bottleneck layer* to reduce the dimensionality accompanied by a *max pooling layer*. The extracted features were fused into the network after *resampling* and *normalization* as a new dimension to the network. The last layer shaped the probabilities of different classes, e.g., surgical proficiency scores or the task categories. The visualization was created in https://netron.app.

- ***LSTM Model***

A recurrent neural network based on LSTM that includes an input layer for the segmented force data ($\boldsymbol{X}_{seg.} \in \mathbb{R}^{S \times C}$), *LSTM layers* with *TanH activation* to interpret the extracted features, a *dropout regularization layer*, a *ReLU activation layer*, and an output layer with *Softmax activation* providing the probability distribution of each surgical task class. The network weights Θ which characterizes the behavior of transformations were identified through nonlinear optimization methods, i.e., *Adam*, to minimize the loss function, e.g., *Categorical Cross-Entropy* and a customized loss function (details in the supplementary codes), in the training data and backpropagation of error throughout the network for updating the weights. The performance of our models was evaluated by generalization through testing on previously unseen data using *accuracy* and *validation loss*. A grid search was applied over the learning rate (between 0.001-0.1), the LSTM unit size (between 100-600), input data window size (between 96-200), and batch size (between 32-128) to tune the hyperparameters. The model architecture visualization is shown in Fig. S6.

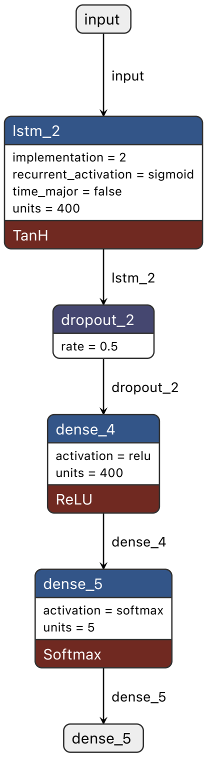

Fig. S6 | The LSTM model architecture used as an experimental model for force profile pattern recognition on time-series of segmented force profiles. The graph shows detailed procedure names and attribute values for task recognition model. Note: the network for skill classification recognition is not included in the report to avoid duplication. The network was comprised of an *LSTM layer* with *TanH activation* to interpret the extracted features, a *dropout regularization layer*, a *ReLU activation layer*, and an output layer with *Softmax activation* providing the probability distribution of each surgical task class. The visualization was created in https://netron.app.

Table S1: List and description of hand-crafted features for the surgical pattern recognition models.

Feature Name	Description
Duration Force	Duration of force application in one task segment
Mean Force	Average of force values in one task segment
Maximum Force	Maximum of force values in one task segment
Minimum Force	Minimum of force values in one task segment
Range Force	Range of force values in one task segment
Median Force	Median of force values in one task segment
Force Standard Deviation	Standard deviation of force values in one task segment
Force Coefficient of Variance	Coefficient of variation of force values in one task segment
Force Distribution Skewness	The extent to which the force data distribution deviates from a normal distribution
Force Distribution Kurtosis	The extent to which the force data distribution is tailed in a normal distribution
Force Distribution Normality Test	Shapiro-Wilk test of normality in force data distribution
Force Profile Peaks Count	Number of force peaks in one task segment
Force Profile Maximum Peak Value	Force peak maximum value in one task segment
Force Time Series Frequency	Dominant time-series harmonics extracted from Fast Fourier Transform (FFT) of force value in one task segment
Force Time Series Period Length	Average time length of force cycles in one task segment
Force 1st Derivative Standard Deviation	Standard deviation for the first derivative of the force signal in one task segment
Force Profile Flat Spots	Maximum run length for each section of force time-series when divided into ten equal-sized intervals
Force Profile Trend Strength	Force time-series trend in one task segment
Force Profile Linearity	Force time-series linearity index (from Teräsvirta's nonlinearity test) in one task segment
Force Profile Stability	Force time-series stability index (variance of the means) in one task segment
Force Profile Lumpiness	Force time-series lumpiness index (variance of the variances) in one task segment
Force Profile Crossing Points	Number of zero crossings in in one task segment
Force Profile Entropy	Force time-series forecastability in one task segment (low values indicate a high signal-to-noise ratio)
Force Profile Heterogeneity	Force time-series heterogeneity in one task segment (based on autoregressive conditional heteroskedasticity (ARCH) effects)
Force Profile Spikiness	Force time series spikiness index (variance of the leave-one-out variances of the remainder component) in one task segment
Force Profile First Autocorrelation Minimum	Time of first minimum of the autocorrelation function in force time-series signal from one task segment
Force Profile First Autocorrelation Zero	Time of first zero crossing of the autocorrelation function in force time-series signal from one task segment
Autocorrelation Function E1	First autocorrelation coefficient from force time-series signal in one task segment
Autocorrelation Function E5	Sum of the first ten squared autocorrelation coefficients from force time-series signal in one task segment

Results

- ***Force Profile Segmentation Model (Fig. 1, Section 2.2)***

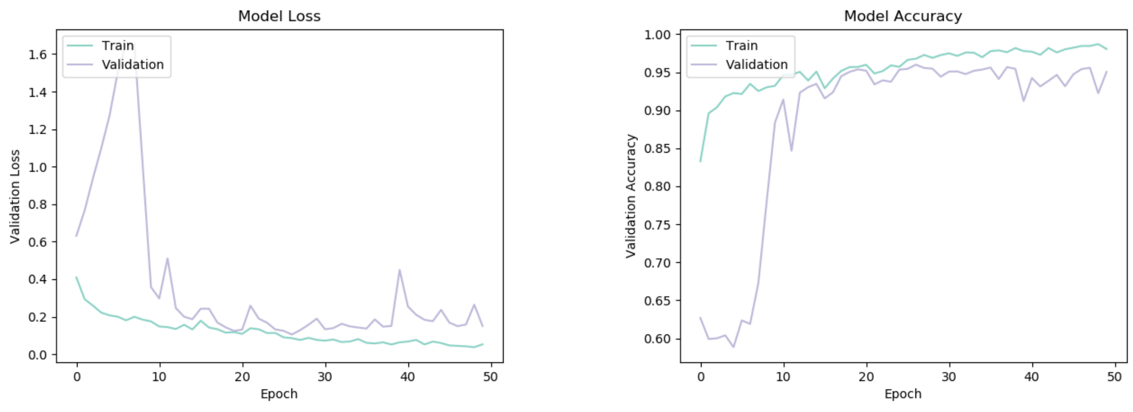

(a) (b)

Fig. S7 | Accuracy and loss function values during training and validation steps for force profile segmentation using U-Net model. History results for model accuracy and loss function value over 50 epochs overlaid for both training and validation iteration. a) The minimum validation loss function value occurred at epoch 27 and was 0.1046 (training loss = 0.0853). b) The historical accuracy for training has a consistent improvement over the trials and achieved 0.98 in training and 0.96 in validation.

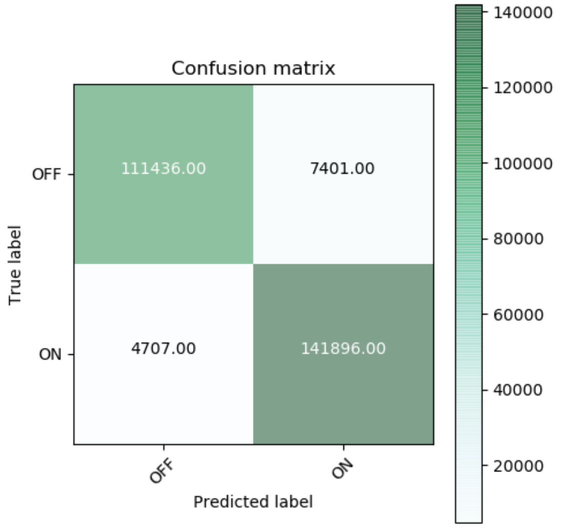

Fig. S8 | The summary prediction results for the segmentation model as binary classification problem using U-Net model. Across the approximately 265K records of force data points with 55.2% in *ON* labels and 44.8% in *OFF* labels after the implementation of our rule-based data balancing, the testing accuracy of classification was 0.9, sensitivity (True Positive Rate: TPR) was 0.97, and specificity (True Negative Rate: TNR) was 0.94.

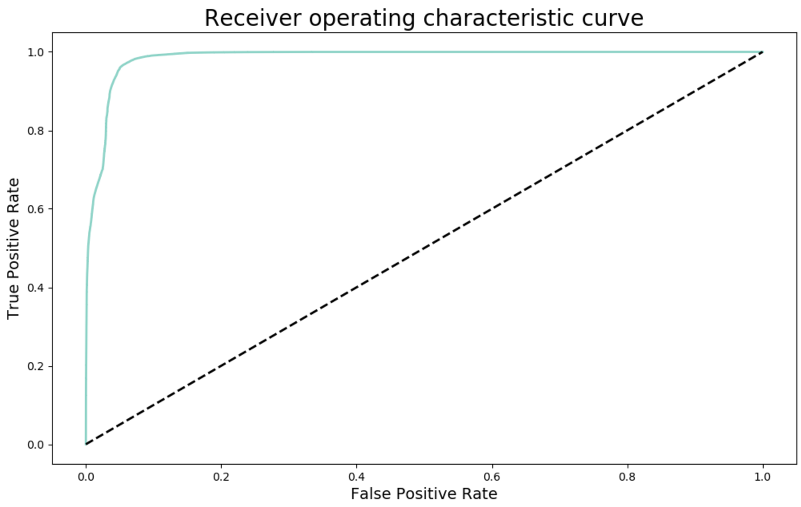

(a)

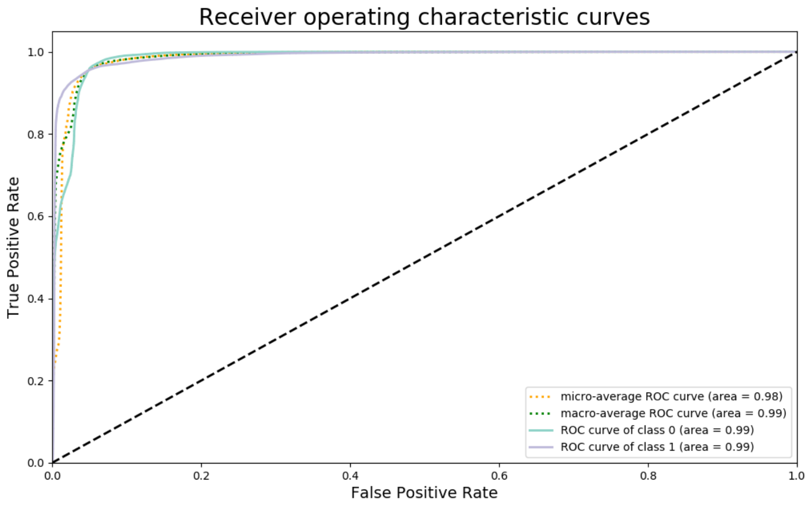

(b)

Fig. S9 | The prediction performance for the segmentation model through receiver operating characteristic curves. The ROC plots illustrate the diagnostic ability of the binary classifier in the segmentation model as the discrimination threshold varies by plotting the TPR (sensitivity or recall) against the FPR (1-specificity). a) Shows the class 0 (*OFF*) ROC curve with AUC value of 0.99. b) Visualizes the class-based comparison of ROC curves along with the macro-average (independently for each class) and micro-average (aggregative contribution for all classes) showing an AUC of 0.99 and 0.98, respectively. Note that One-vs-One and One-vs-Rest class AUC has identical results given the 2-class problem in hand.

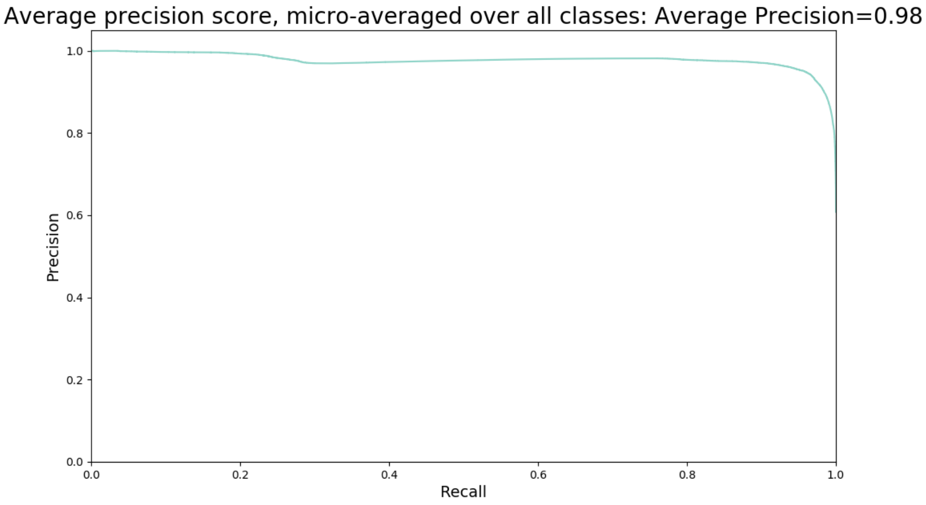

(a)

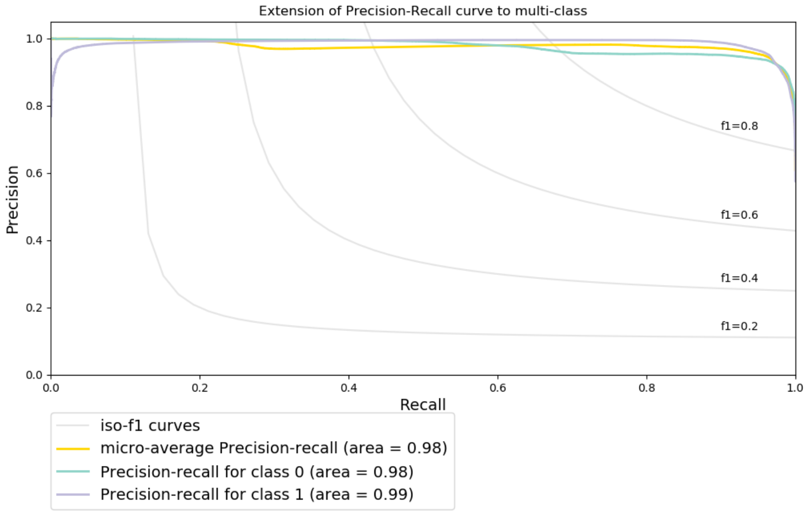

(b)

Fig. S10 | Illustrating the trade-off between true positive rate and positive predictive value in different probability thresholds in segmentation. a) Micro-averaged (aggregative contribution for all classes) precision-recall score (area under the curve) for both classes were 0.98. b) The precision-recall value was 0.99 for the class of forceps *ON* (class 1) and 0.98 for *OFF* (class 0). The inclusion of ISO-F1 curves shows that for all the points in the precision/recall space, most instances have F1 scores over 0.8 in the classification problem.

- ***Surgical Skill Classification Model (Fig. 1, Section 2.3)***

  - ***XGBoost Model***

    A baseline model using XGBoost which allows parallel computation was implemented for skill classification. A regularization parameter *lambda* was used to avoid overfitting by limiting the gain and similarity score of the model tree nodes. Tree pruning was applied using *cover*, i.e., minimum allowed child node weight (*minimum child weight*). In addition, *learning rate* (i.e., *eta*) was used as another regularization parameter with lower values reducing the possibility of overfitting. The value of *eta* determines the shrinkage of the contribution of a tree at each boosting iteration. We changed *learning rate* values from 0.01 to 0.45 with intervals of 0.05 and *minimum child weight* within [0.0-4.0] with an increment of 0.5. Grid search was applied on the 5-fold cross-validated scores of the XGBoost model. Feature selection was implemented through importance ranking (Force 1st Derivative Standard Deviation, Minimum Force, Force Profile Crossing Points, and Distribution Normality Test was selected based on a score threshold of 0.05). The testing accuracy of model with the best hyperparameters (i.e., *minimum child weight* = l.0 and *learning rate* = 0.06) was 0.65.

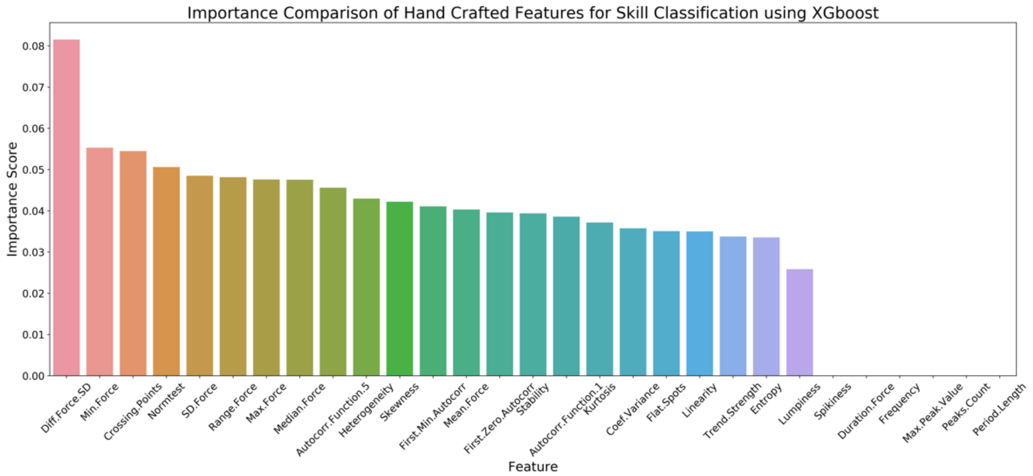

Fig. S11 | Importance ranking of the hand-crafted features in skill classification using XGBoost modeling. Force 1st Derivative Standard Deviation, Minimum Force, Force Profile Crossing Points, and Distribution Normality Test were selected based on a score threshold of 0.05.

Table S2 | Relative importance scores of features for skill classification with a threshold of 0.05 using XGBoost model.

**Feature**	**Importance Score**
Force 1st Derivative Standard Deviation	0.082
Minimum Force	0.055
Force Profile Crossing Points	0.054
Distribution Normality Test	0.051

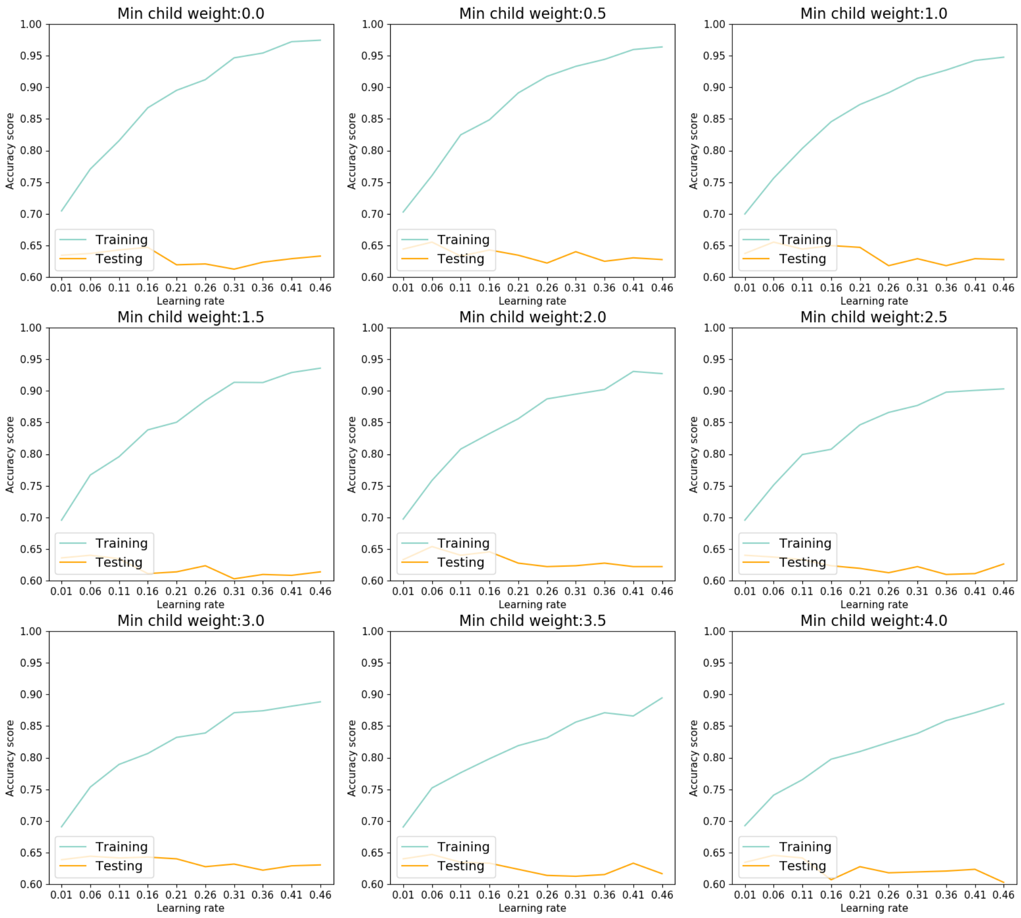

Fig. S12 | Experimental results of skill classification with various learning rate and minimum child weight using XGBoost model. Grid search over the hyperparameters could reduce the gap between training and testing accuracies thus the overfitting. The testing accuracy of model with the best hyperparameters (i.e., *minimum child weight* = 1.0 and *learning rate* = 0.06) was 0.65.

Statistical Explanation on Feature Selection: A feature with more relevance and greater contribution to the performance of the ML model has higher importance ranking. The reason can be discussed in the context of their statistical analysis and significance. In skill classification, some features showed a significant difference between skill classes in two-way ANOVA tests (Force Profile First Autocorrelation Zero: *p-value* < 0.001; Minimum Force: *p-value* < 0.001; Median Force: *p-value* < 0.001; Distribution Normality Test: *p-value* < 0.001; Force Profile Flat Spots: *p-value* < 0.001; Force Profile Linearity: *p-value* = 0.02). Experts applied less forces than the *Novice* group (Mean of Median Force 0.07N vs. 0.23N). Additionally, *Novices* had more Force Profile Flat Spots (Mean 20.64 vs. 16.52). Compared to Experts, Novices tend to exhibit hesitancy and perhaps unsafe force application behavior when using SmartForceps. According to the Distribution Normality Test for Experts, forces within this group were more consistent (Mean 0.85 vs. 0.81).

- ***KNN Permutation Feature Importance Ranking***

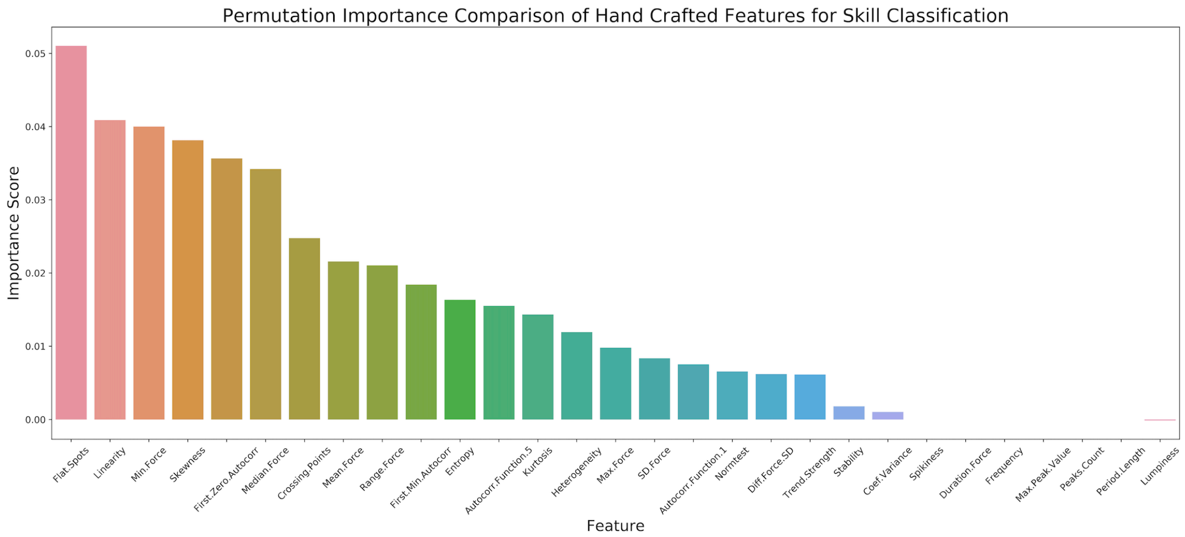

Fig. S13 | Permutation importance ranking of the hand-crafted features in skill classification using KNN modeling. Force Profile Flat Spots, Force Profile Linearity, Minimum Force, Force Distribution Skewness, Force Profile First Autocorrelation Zero, and Median Force were selected based on a score threshold of 0.03.

Table S3 | Relative importance scores of features for skill classification with a threshold of 0.03 using KNN Model.

**Feature**	**Importance Score**
Force Profile Flat Spots	0.051
Force Profile Linearity	0.041
Minimum Force	0.040
Force Distribution Skewness	0.038
Force Profile First Autocorrelation Zero	0.036
Median Force	0.034

Selected Features for Skill Classification Model:

- Force 1st Derivative Standard Deviation
- Minimum Force
- Force Profile Crossing Points
- Distribution Normality Test
- Force Profile Flat Spots
- Force Profile Linearity
- Force Distribution Skewness
- Force Profile First Autocorrelation Zero
- Median Force

○ ***FTFIT Model***

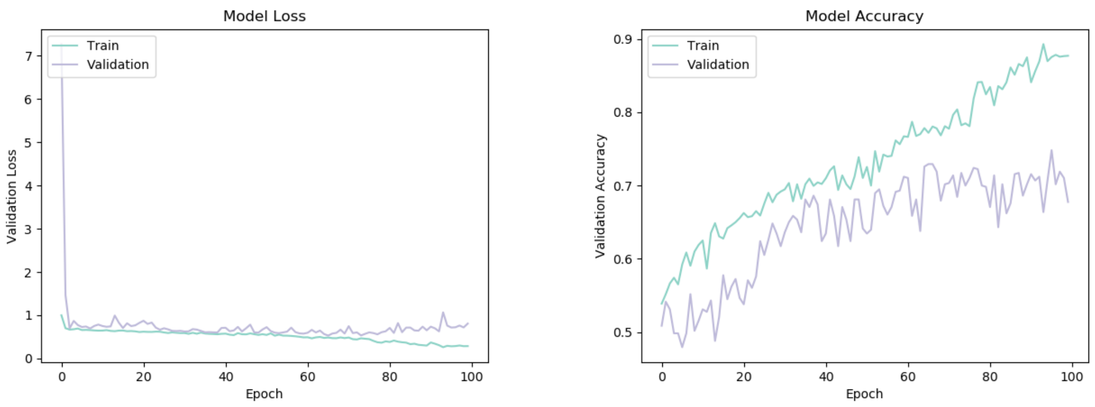

(a) (b)

Fig. S14 | Accuracy and loss function values during training and validation steps for force profile surgical skill pattern recognition using FTFIT model. History results for model accuracy and loss function value over 100 epochs overlaid for both training and validation iteration. a) The minimum validation loss function value occurred at epoch 66 and was 0.5285 (training loss = 0.4841). b) The historical accuracy for training has a consistent improvement over the training trials but becomes steady after around epoch 60 indicating an overfitting situation (achieved 0.88 in training and 0.75 in validation). To avoid overfitting, early stopping was implemented considering the minimum validation loss value at epoch 66.

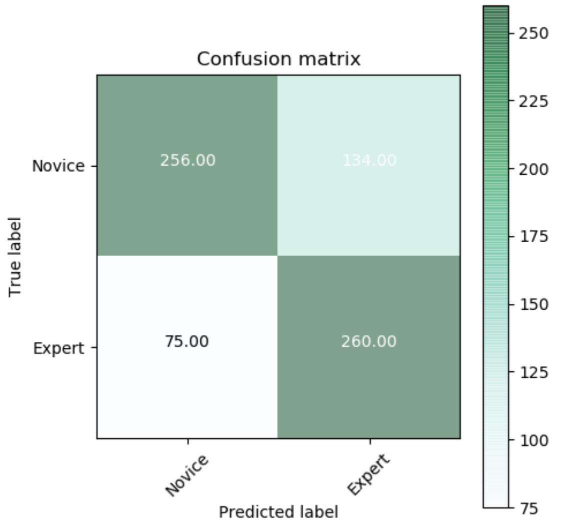

Fig. S15 | The summary prediction results for surgical skill prediction using FTFIT model. Across the 725 records of force segments with 46.2% in *Expert* labels and 53.8% in *Novice* labels, classification accuracy was 0.71, sensitivity (True Positive Rate: TPR) was 0.78, and specificity (True Negative Rate: TNR) was 0.66.

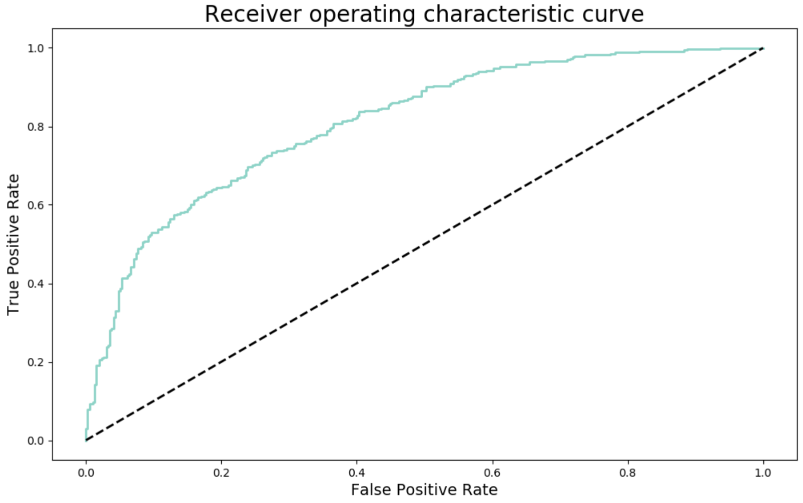

(a)

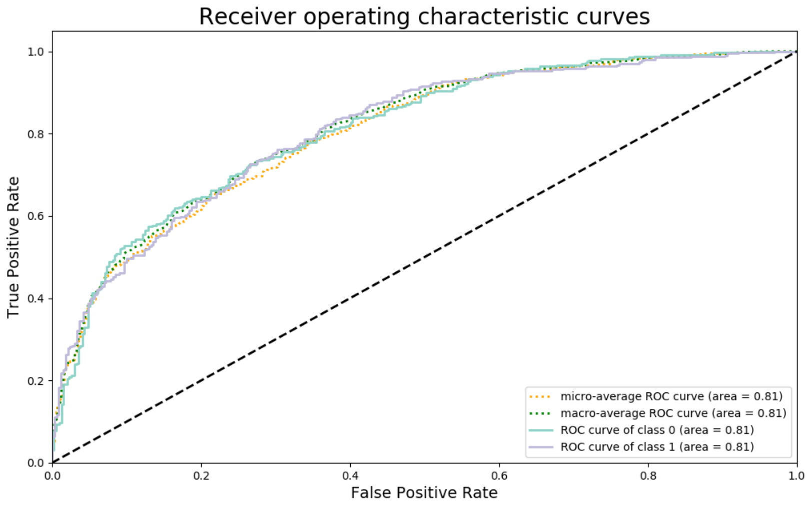

(b)

Fig. S16 | The prediction performance for the skill classification model through receiver operating characteristic curves. The ROC plots illustrate the diagnostic ability of the binary classifier in the skill classification model as the discrimination threshold varies by plotting the TPR (sensitivity or recall) against the FPR (1-specificity). a) Shows the class 0 (*Novice*) ROC curve with AUC value of 0.81. b) Visualizes the class-based comparison of ROC curves along with the macro-average (independently for each class) and micro-average (aggregative contribution for all classes) showing an AUC of 0.81 in both settings. Note that One-vs-One and One-vs-Rest class AUC has identical results given the 2-class problem in hand.

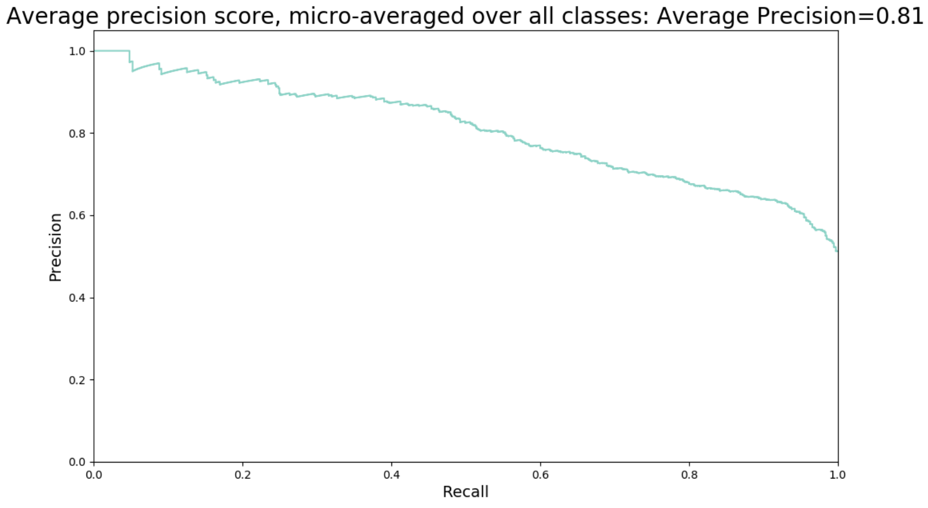

(a)

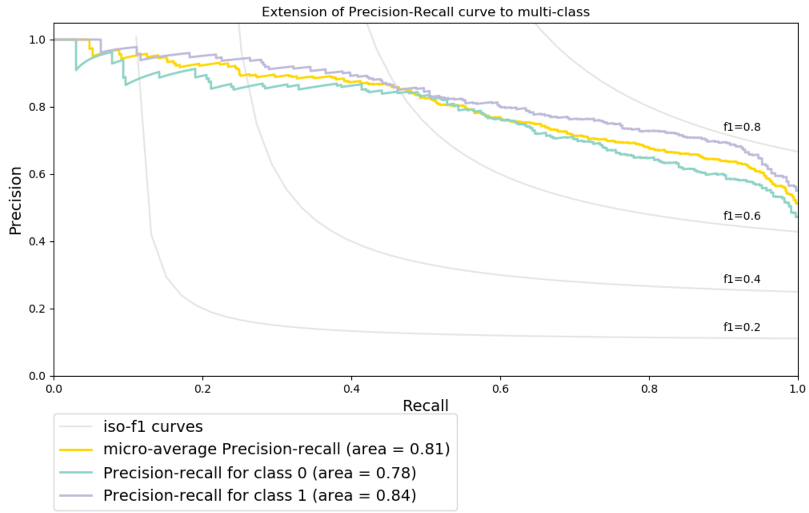

(b)

Fig. S17 | Illustrating the trade-off between true positive rate and positive predictive value in different probability thresholds in the skill classification. a) Micro-averaged (aggregative contribution for all classes) precision-recall score (area under the curve) for both classes were 0.81. b) The scores for each individual class of *Novice* (class 0) and *Expert* (class 1) were 0.78 and 0.84, respectively. The inclusion of ISO-F1 curves show that for all the points in the precision/recall space, almost half of the instances have F1 scores over 0.6 in the classification problem.

- ***Surgical Task Recognition Model (Fig. 1, Section 2.3)***

  - ***XGBoost Model***

    A baseline model using XGBoost was used for task recognition similar to skill classification. Grid search was applied on the 5-fold cross-validated scores of the XGBoost model. Feature selection was implemented through importance ranking (Force Profile Entropy, Median Force, Force Profile Trend Strength, First Autocorrelation Minimum, and Force Profile Crossing Points was selected based on a score threshold of 0.05). The testing accuracy of model with the best hyperparameters (i.e., *minimum child weight* = 2.0 and *learning rate* = 0.01) was 0.81.

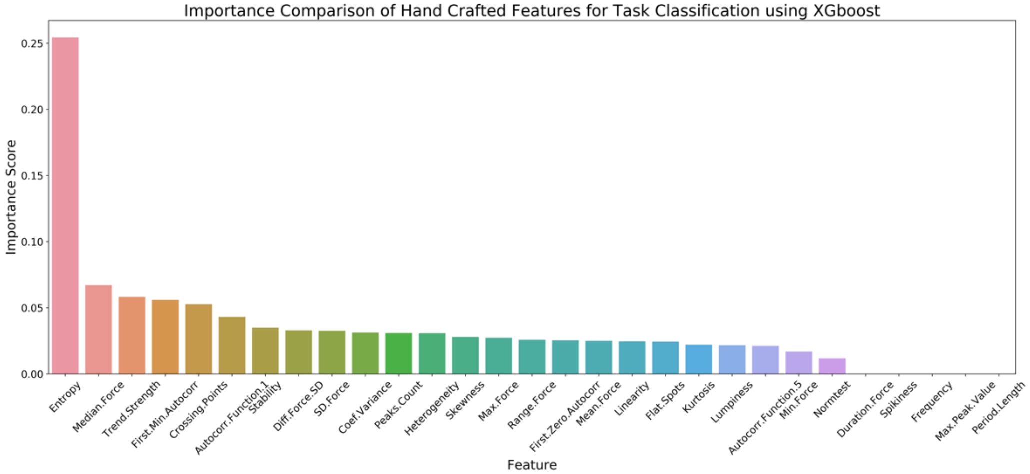

Fig. S18 | Importance ranking of the hand-crafted features in task recognition using XGBoost modeling. Force Profile Entropy, Median Force, Force Profile Trend Strength, Force Profile First Autocorrelation Minimum, Force Profile Crossing Points were selected based on a score threshold of 0.05.

Table S4 | Relative importance scores of features for task recognition with a threshold of 0.05 using XGBoost model.

**Feature**	**Importance Score**
Force Profile Entropy	0.254
Median Force	0.067
Force Profile Trend Strength	0.058
Force Profile First Autocorrelation Minimum	0.056
Force Profile Crossing Points	0.053

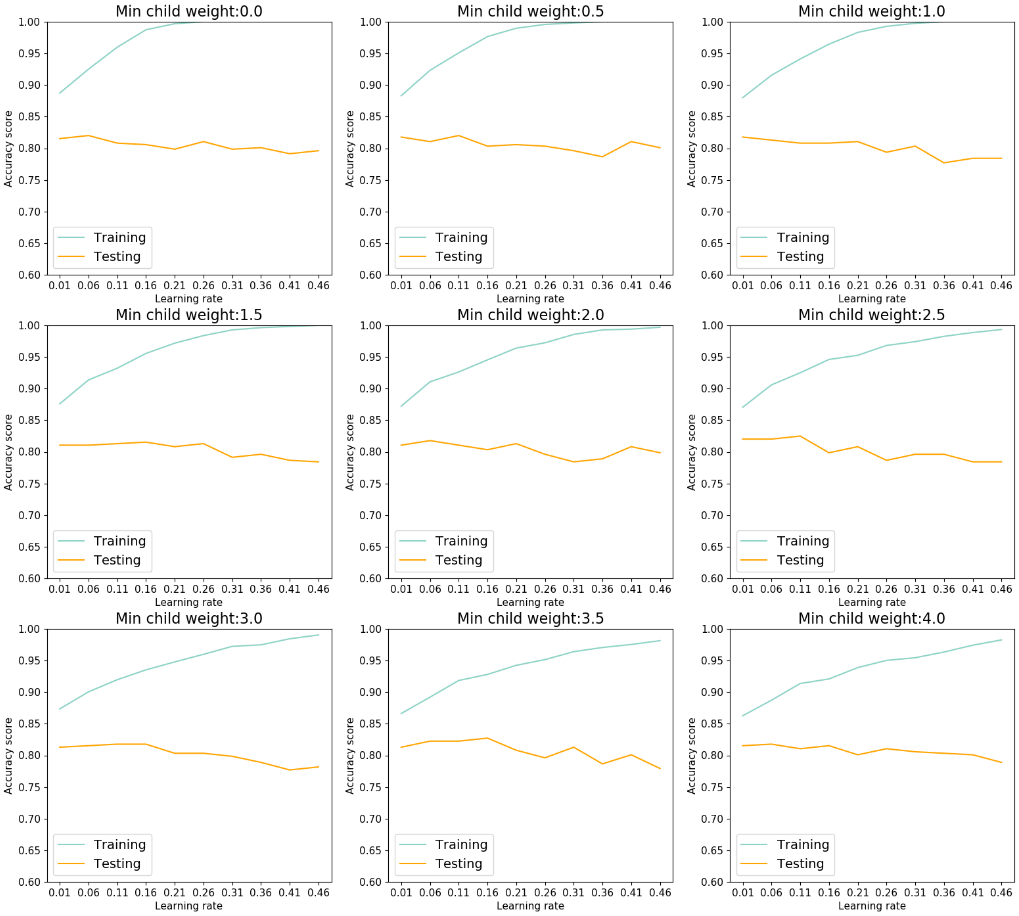

Fig. S19 | Experimental results of task recognition with various learning rate and minimum child weight using XGBoost model. Grid search over the hyperparameters could reduce the gap between training and testing accuracies thus the overfitting. The testing accuracy of model with the best hyperparameters (i.e., *minimum child weight* = 2.0 and *learning rate* = 0.01) was 0.81.

Statistical Explanation on Feature Selection: A feature with more relevance and greater contribution to the performance of the ML model has higher importance ranking. The reason can be discussed in the context of their statistical analysis and significance. In task recognition, some features showed a significant difference between task classes in two-way ANOVA tests (Entropy: *p-value* < 0.001; Median Force: *p-value* < 0.001; Minimum Force: *p-value* < 0.001; Force Profile Trend Strength: *p-value* < 0.001; Force Profile First Autocorrelation Minimum: *p-value* < 0.001; Force Profile First Autocorrelation Zero: *p-value* < 0.001; Force Profile Flat Spots: *p-value* < 0.001) [1]. As a result, the Entropy values for *Coagulation* (Mean 0.72) were significantly higher than the *non-Coagulation* tasks (Mean 0.65), indicating its complexity and diversity in a variety of situations and tissues. This point was also reflected in Force Profile Flat Spots (Mean *Coagulation* 12.86 vs. *non-Coagulation* 20.01) and Force Profile Trend Strength (Mean *Coagulation* 0.68 vs. *non-Coagulation* 0.89). The magnitude-related features, e.g., Median Force (Mean *Coagulation* -0.02N vs. *non-Coagulation* 0.19N), were also a defining factor, as surgical tasks differed from *Coagulation* in their nature and force application spots.

- ***KNN Permutation Feature Importance Ranking***

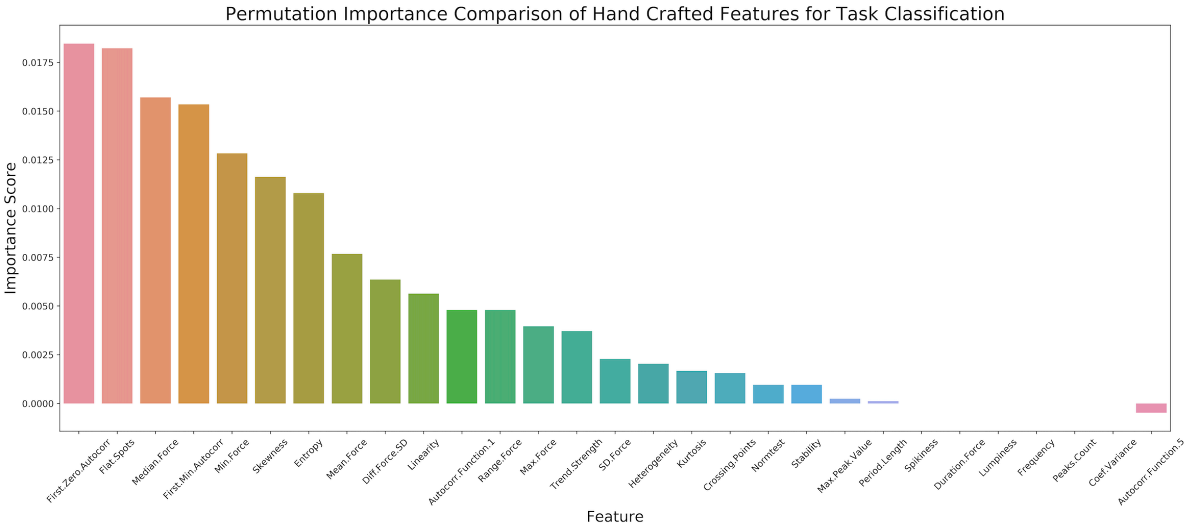

Fig. S20 | Permutation importance ranking of the hand-crafted features in task recognition using KNN modeling. Force Profile First Autocorrelation, Force Profile Flat Spots, Median Force, and Force Profile First Autocorrelation Minimum were selected based on a score threshold of 0.015.

Table S5 | Relative importance scores of features for task recognition with a threshold of 0.015 using KNN model.

**Feature**	**Importance Score**
Force Profile First Autocorrelation Zero	0.018
Force Profile Flat Spots	0.018
Median Force	0.016
Force Profile First Autocorrelation Minimum	0.015

Selected Features for Task Recognition Model:

- Force Profile Entropy
- Median Force
- Force Profile Trend Strength
- Force Profile First Autocorrelation Minimum
- Force Profile Crossing Points
- Force Profile First Autocorrelation Zero
- Force Profile Flat Spots

- ***FTFIT Model***

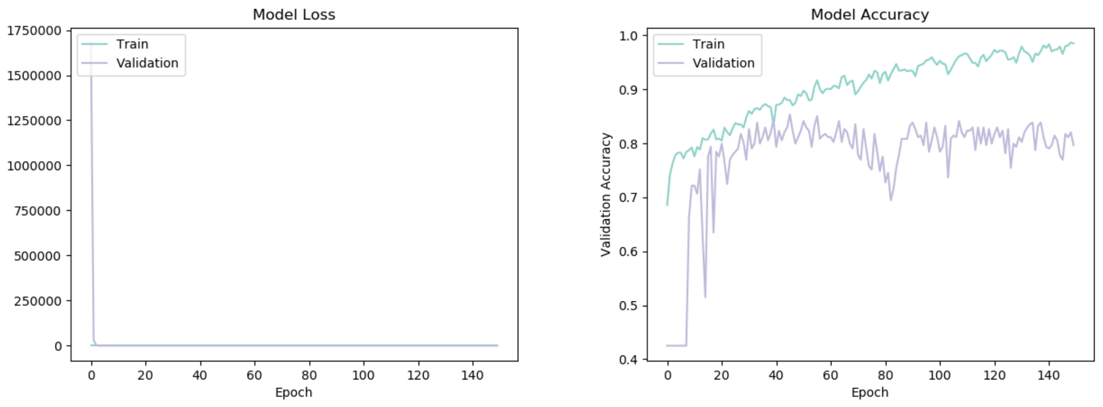

(a) (b)

Fig. S21 | Accuracy and loss function values during training and validation steps for force profile surgical task pattern recognition using FTFIT model. History results for model accuracy and loss function value over 150 epochs overlaid for both training and validation iteration. a) The minimum validation loss function value occurred at epoch 46 and was 0.4002 (training loss = 0.3025). b) The historical accuracy for validation has a consistent improvement over the training trials but becomes steady after around epoch 40 indicating an overfitting situation (achieved 0.99 in training and 0.82 in validation). To avoid overfitting, early stopping was implemented considering the minimum validation loss value at epoch 46.

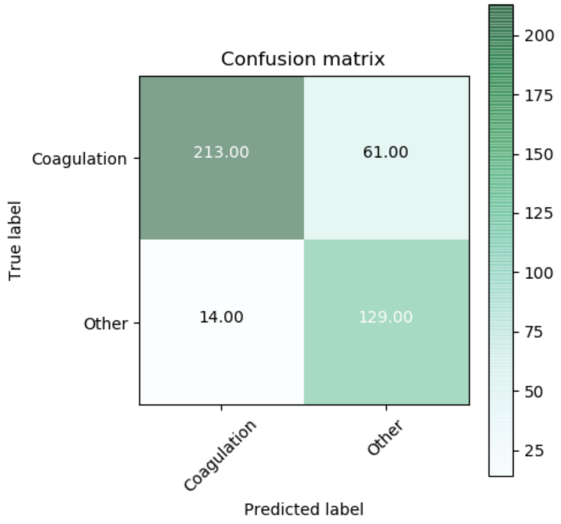

Fig. S22 | The summary prediction results for surgical task recognition using FTFIT model. Across the 417 records of force segments with 65.7% in coagulation and 34.3% in non-coagulation tasks, the average accuracy of classification was 0.82, sensitivity (True Positive Rate: TPR) was 0.90, and specificity (True Negative Rate: TNR) was 0.78.

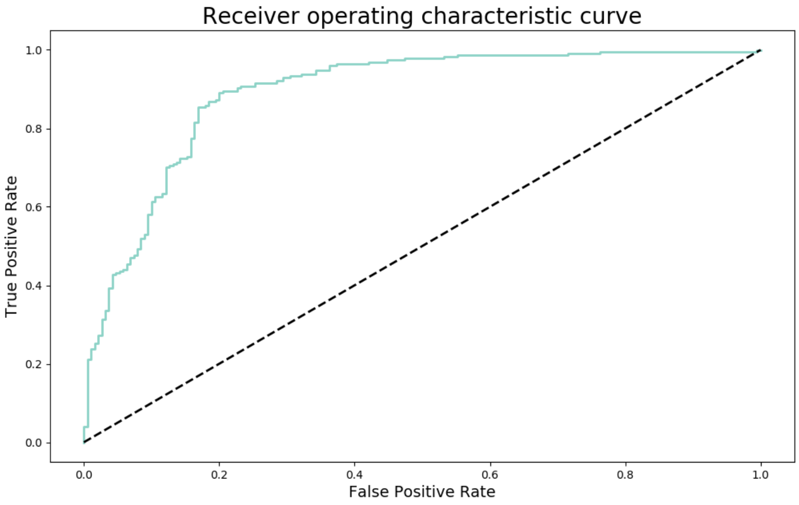

(a)

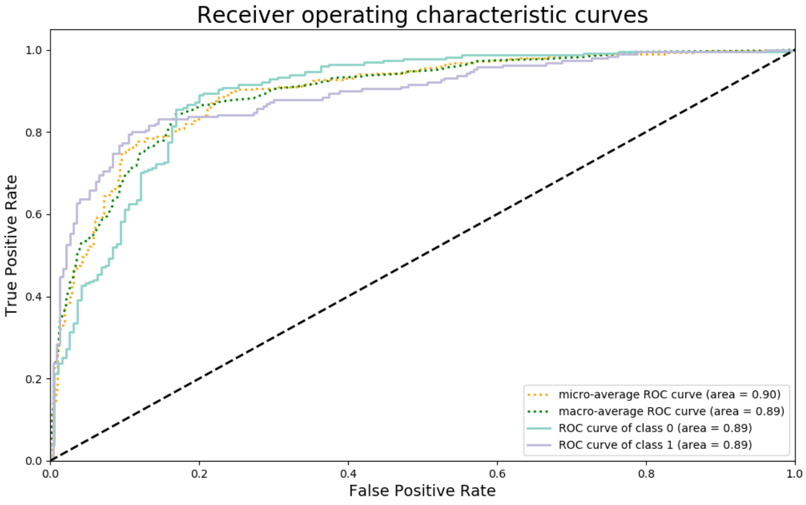

(b)

Fig. S23 | The prediction performance for the task recognition model through receiver operating characteristic curves. The ROC plots illustrate the diagnostic ability of the classifier in the task recognition model as the discrimination threshold varies by plotting the TPR (sensitivity or recall) against the FPR (1-specificity). a) Shows the class 0 (*Coagulation*) ROC curve with AUC value of 0.89. b) Visualizes the class-based comparison of ROC curves along with the macro-average One-vs-One and One-vs-Rest comparisons (independently for each class) as well as the micro-average values (aggregative contribution for all classes) which were equal to 0.89 and 0.90, respectively.

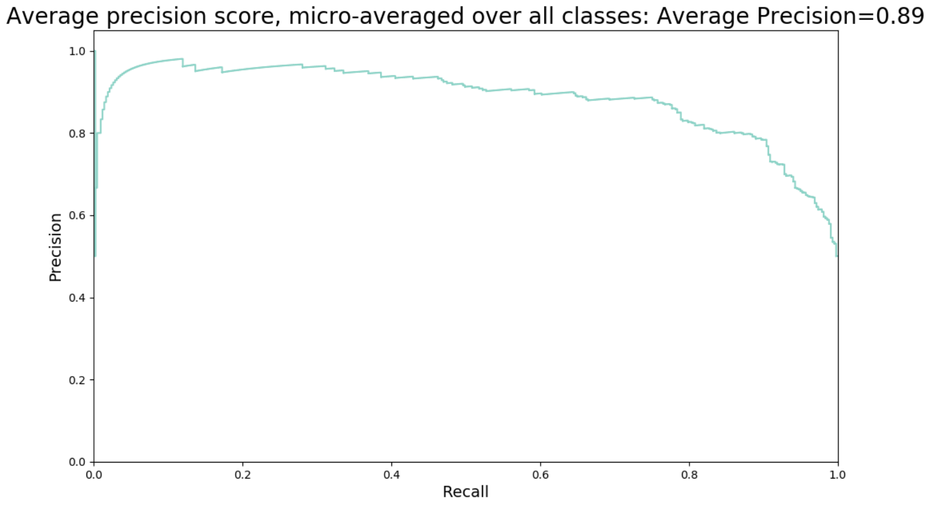

(a)

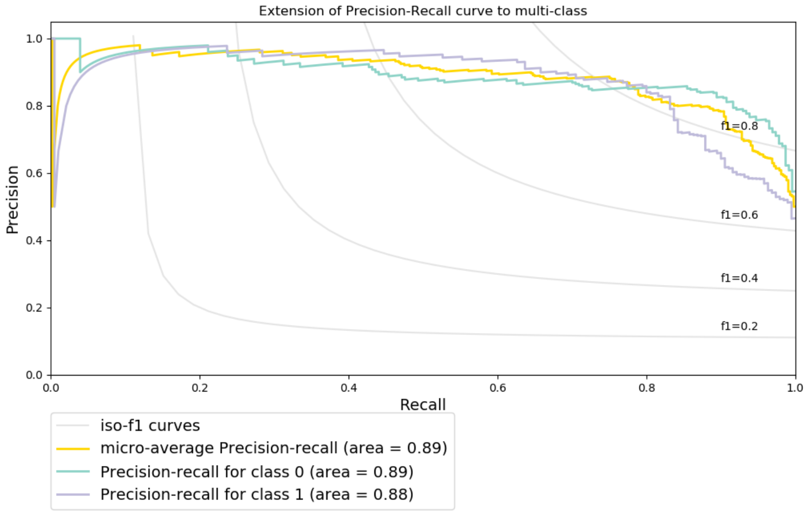

(b)

Fig. S24 | Illustrating the trade-off between true positive rate and positive predictive value in different probability thresholds in the task recognition. a) Micro-averaged (aggregative contribution for all classes) precision-recall score (area under the curve) for all classes was 0.89. b) The inclusion of ISO-F1 curves show that for all the points in the precision/recall space, most of the instances for all classes have F1 scores over 0.6 in the classification problem. The precision-recall score for each class was: *Coagulation* (class 0) = 0.89 and *non-Coagulation* (class 1) = 0.88.

Glossary

Bipolar forceps: A medical device with two "active" electrodes, i.e., forceps**,** for sealing blood vessels.

Cavernous angioma: A type of venous malformation due to endothelial dysmorphogenesis from a lesion which is present at birth.

Coagulation: Cessation of blood loss from a damaged vessel in surgery.

Dissection: Cutting or separation of tissues in surgery.

FTFIT: Force Time-series Feature-based InceptionTime.

Glioma: A type of tumor that starts in the glial cells of the brain or the spine.

Hemangioblastoma: A type of vascular tumor of the central nervous system that originate from the vascular system, usually during middle age.

HIPAA: Health Insurance Portability and Accountability Act.

Intracranial pathology: Any hemorrhage, new hydrocephalus, cerebral edema, tumor, abscess, or ischemic stroke within the skull dated in the previous 7 days by clinical findings and confirmed by head CT.

LSTM: Long Short-Term Memory.

Manipulating: Moving cotton or other non-tissue objects in surgery.

Meningioma: A typically a slow-growing tumor that forms from the meninges, the membranous layers surrounding the brain and spinal cord.

PIPEDA: Personal Information Protection and Electronic Documents Act.

Pulling: Moving and retaining tissues in one direction in surgery.

Retracting: Grasping and retaining tissue for surgical exposure in surgery.

Schwannoma: A usually benign nerve sheath tumor composed of Schwann cells, which normally produce the insulating myelin sheath covering peripheral nerves.

SmartForceps: A sensorized bipolar forceps coupled to an intelligent software platform.

Trigeminal neuralgia/hemifacial spasm: Two conditions affecting nerves in the face. Symptoms of trigeminal neuralgia include short episodes of sharp facial pain. Hemifacial spasm, while painless, may cause severe twitching of the facial muscles.

Tumor resection: Removing a tumor as a common type of cancer surgery.

References

1. Baghdadi, A., et al., *A data-driven performance dashboard for surgical dissection.* Scientific Reports, 2021. **11**(1): p. 1-13.
